# A Network Biology Approach Identifies Molecular Cross-Talk between Normal Prostate Epithelial and Prostate Carcinoma Cells

**DOI:** 10.1371/journal.pcbi.1004884

**Published:** 2016-04-28

**Authors:** Victor Trevino, Alberto Cassese, Zsuzsanna Nagy, Xiaodong Zhuang, John Herbert, Philipp Antzack, Kim Clarke, Nicholas Davies, Ayesha Rahman, Moray J. Campbell, Michele Guindani, Roy Bicknell, Marina Vannucci, Francesco Falciani

**Affiliations:** 1 Catedra de Bioinformatica, Escuela de Medicina, Tecnologico de Monterrey, Monterrey, Nuevo Leon, Mexico; 2 Department of Methodology and Statistics, Maastricht University, Maastricht, Netherlands; 3 School of Experimental and Clinical Medicine, University of Birmingham, Edgbaston, Birmingham, United Kingdom; 4 School of Immunity and Infection, University of Birmingham, Edgbaston, Birmingham, United Kingdom; 5 Institute of Integrative Biology, University of Liverpool, Liverpool, United Kingdom; 6 School of Cancer Sciences, College of Medicine and Dentistry, University of Birmingham, Edgbaston, Birmingham, United Kingdom; 7 School of Pharmacy, Faculty of Science and Engineering, University of Wolverhampton, Wolverhampton, United Kingdom; 8 Department of Pharmacology and Therapeutics, Roswell Park Cancer Institute, Buffalo, New York, United States of America; 9 Department of Biostatistics, University of Texas MD Anderson Cancer Center, Houston, Texas, United States of America; 10 Department of Statistics, Rice University, Houston, Texas, United States of America; H.Lee Moffitt Cancer Center and Research Institute, UNITED STATES

## Abstract

The advent of functional genomics has enabled the genome-wide characterization of the molecular state of cells and tissues, virtually at every level of biological organization. The difficulty in organizing and mining this unprecedented amount of information has stimulated the development of computational methods designed to infer the underlying structure of regulatory networks from observational data. These important developments had a profound impact in biological sciences since they triggered the development of a novel data-driven investigative approach. In cancer research, this strategy has been particularly successful. It has contributed to the identification of novel biomarkers, to a better characterization of disease heterogeneity and to a more in depth understanding of cancer pathophysiology. However, so far these approaches have not explicitly addressed the challenge of identifying networks representing the interaction of different cell types in a complex tissue. Since these interactions represent an essential part of the biology of both diseased and healthy tissues, it is of paramount importance that this challenge is addressed. Here we report the definition of a network reverse engineering strategy designed to infer directional signals linking adjacent cell types within a complex tissue. The application of this inference strategy to prostate cancer genome-wide expression profiling data validated the approach and revealed that normal epithelial cells exert an anti-tumour activity on prostate carcinoma cells. Moreover, by using a Bayesian hierarchical model integrating genetics and gene expression data and combining this with survival analysis, we show that the expression of putative cell communication genes related to focal adhesion and secretion is affected by epistatic gene copy number variation and it is predictive of patient survival. Ultimately, this study represents a generalizable approach to the challenge of deciphering cell communication networks in a wide spectrum of biological systems.

## Author Summary

In the current era of cancer research, stimulated by the release of the entire human genome, it has become increasingly clear that to understand cancer we need to understand how the many thousands of genes and proteins involved interact. Modern techniques have enabled the collection of unprecedented amounts of high quality data describing the state of these molecules during cancer development. In cancer research particularly, this strategy has been particularly successful, leading to the discovery of new drugs able to target key factors promoting cancer growth. However, a large body of research suggests that in complex organs, the interaction between cancer and its surrounding environment is an essential part of the biology of both diseased and healthy tissues, therefore it is of paramount importance that this process is further investigated. Here we report a strategy designed to reveal communication signals between cancer cells and adjacent cell types. We apply the strategy to prostate cancer and find that normal cells surrounding the tumour do exert an anti-tumour activity on prostate cancer cells. By using a statistical model which integrates multiple levels of genetic data, we show that cell-to-cell communication genes are controlled by DNA alterations and have potential prognostic value.

## Introduction

Prostate Cancer is the most common cancer in males. It is characterized by a considerable molecular and phenotypic heterogeneity that results in radically different clinical outcomes [1].

The role of tumour microenvironment in the development of cancer is crucial. More specifically, the expression of growth and motility factors, extracellular matrix components produced by stromal cells, is linked to the pathophysiology of the tumour and it often predictive of clinical outcome. Stromal cells, such as fibroblasts and endothelial cells secrete many factors that influence the expansion of the tumour. For example, they secrete most of the enzymes involved in extracellular matrix breakdown and produce growth factors that control tumour cell proliferation, apoptosis, and migration [2]. They also secrete pro-inflammatory cytokines, which play a major role in a wide spectrum of pathophysiology mechanisms (e.g. chemo attraction, neoplastic transformation, angiogenesis, tumour clonal expansion and growth, passage through the ECM, intravasation into blood or lymphatic vessels and the non-random homing of tumour metastasis to specific sites) [3]. In addition to tumour promoting factors, they also secrete tumour suppressor factors that can potentially have an anti-tumour effect on adjacent tumour cells [4]. Current research on the role of stroma is principally focused on immune cells fibroblasts and cells of the vasculature such as endothelial cells. However, since other cell types, such as normal epithelial cells, also produce a number of these factors, such as IL-6 [5], TNFα [6] [7] and TGFβ1 [7] it is reasonable to hypothesize that they may also play an important role in influencing the molecular and physiological state of tumour cells.

The intricacy in the biology of cell-to-cell communication and the relatively small amount of available knowledge makes understanding the biological networks underlying the development of tumour microenvironment a suitable challenge for a systems-level approach. The powerful combination of functional genomics and computational biology have contributed to the discovery of novel signaling networks in the biology of cancer [8] [9], including cell communication networks [10]. However, so far there has been no attempt to develop a completely data-driven systems biology approach to discover novel cell-communication networks.

Here we describe a data-driven strategy we developed to address this challenge. Our approach is designed to “learn” the underlying structure of cell-to-cell communication networks from functional genomics datasets, representing the transcriptional state of normal and adjacent tumour cells.

The application of this novel analysis strategy to prostate cancer revealed genes whose expression is associated to directional signals linking normal and tumour epithelial cells. Remarkably, experimental validation of our predictions using an in vitro co-culture system recapitulated the predicted transcriptional response and revealed that normal epithelial cells have the potential to revert some of the phenotypic traits of tumour cells. Moreover, by integrating genetics, gene expression and tumour features in a single conceptual model, we were able to show that putative cell communication networks, involved in focal adhesion and protein secretion are perturbed by genetic mutations and that are linked to survival.

Ultimately, the experimental validation of the hypothesis generated from the model support the approach we have developed, which explicitly search for candidate directional signals between different cell types. Its application to a wider range of biological systems is likely to have a profound impact in the field of functional genomics.

## Results

### Overview of the analysis and validation strategy

**Fig 1:**
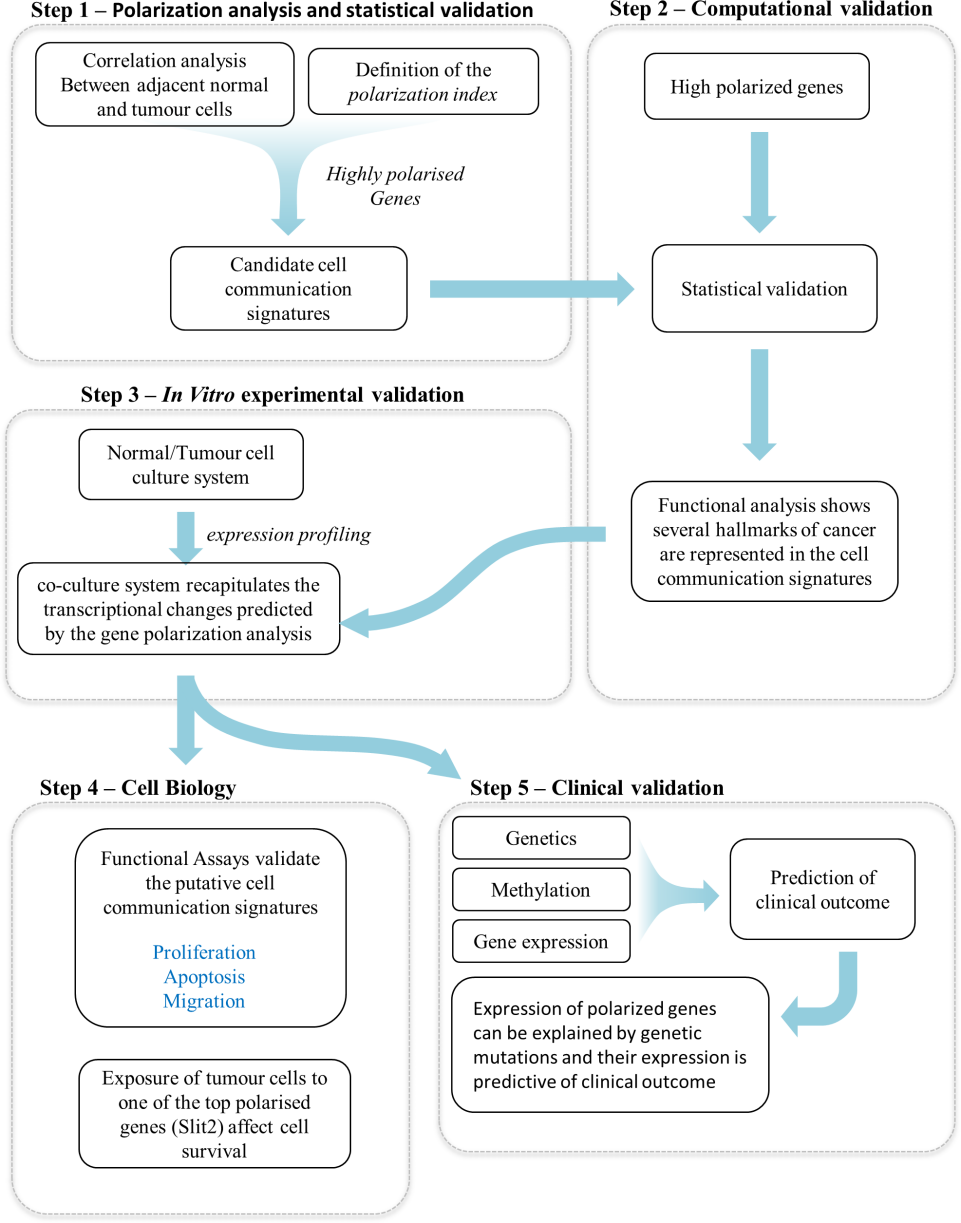
Overview of the analysis and validation strategy.

Our study is based on a data analysis workflow which includes reverse engineering techniques to identify gene expression signatures that may be involved in cell to cell communications. The strategy we followed, which is summarized in **Fig 1,** is based on several cycles of computational analysis, hypothesis generation and experimental validation. The workflow consisted of five distinct but interconnected steps.

#### Step 1

Correlation analysis of a gene expression profiling dataset linking genes expressed in normal and tumor epithelial cells. The analysis of the resulting network with a novel topological index (*polarization index*) identified candidate genes involved in cell-to-cell communication.

#### Step 2

The application of two different statistical approaches shows that the cell communication candidate genes represent gene signatures that are statistically robust, such that they could not be generated by random chance or by issues of sample purity. Moreover, functional analysis shows that candidate genes are enriched in functional terms representing multiple hallmarks of cancer.

#### Step 3

Experimental validation: Expression profiling. By using a transwell based cell co-culture system we show that changes in gene expression in normal and tumor cells induced by co-culture recapitulate the signatures predicted by co-expression analysis and representing multiple hallmarks of cancer.

#### Step 4

Experimental validation: Cell Biology. By using a transwell based cell co-culture system in conjunction with several cell biology assays we show that the presence of normal cells decreases the aggressiveness of tumor cells and that such reduction in survival can be reproduced in tumor cells by exposing them to a recombinant protein encoded by one of the most statistically significant cell communication gene candidates,

#### Step 5

Clinical validation. By using a publicly available clinical study, including multilevel genomics and functional genomics data as well as clinical outcome, we prove that the expression of a significant number of cell communication candidates identified by our procedure can be explained by methylation or correlate with genetic mutations and are predictive of clinical outcome. We also show that the expression of these genes is higher in normal tissue with respect to adjacent normal and tumor tissue.

### A gene co-expression network reveal transcriptional signatures linking adjacent tumour and normal epithelial cells

The overarching goal of this project was to develop a data driven strategy to identify molecular pathways involved in cell-to-cell crosstalk. We first set to test whether gene expression profiles across normal samples may correlate with the gene expression profiles from the matching tumour samples. We reasoned that if such correlated profiles exist they might be a manifestation of the signaling events between normal and tumour epithelial cells and may shed new light on the role of normal epithelia in prostate cancer.

**Fig 2:**
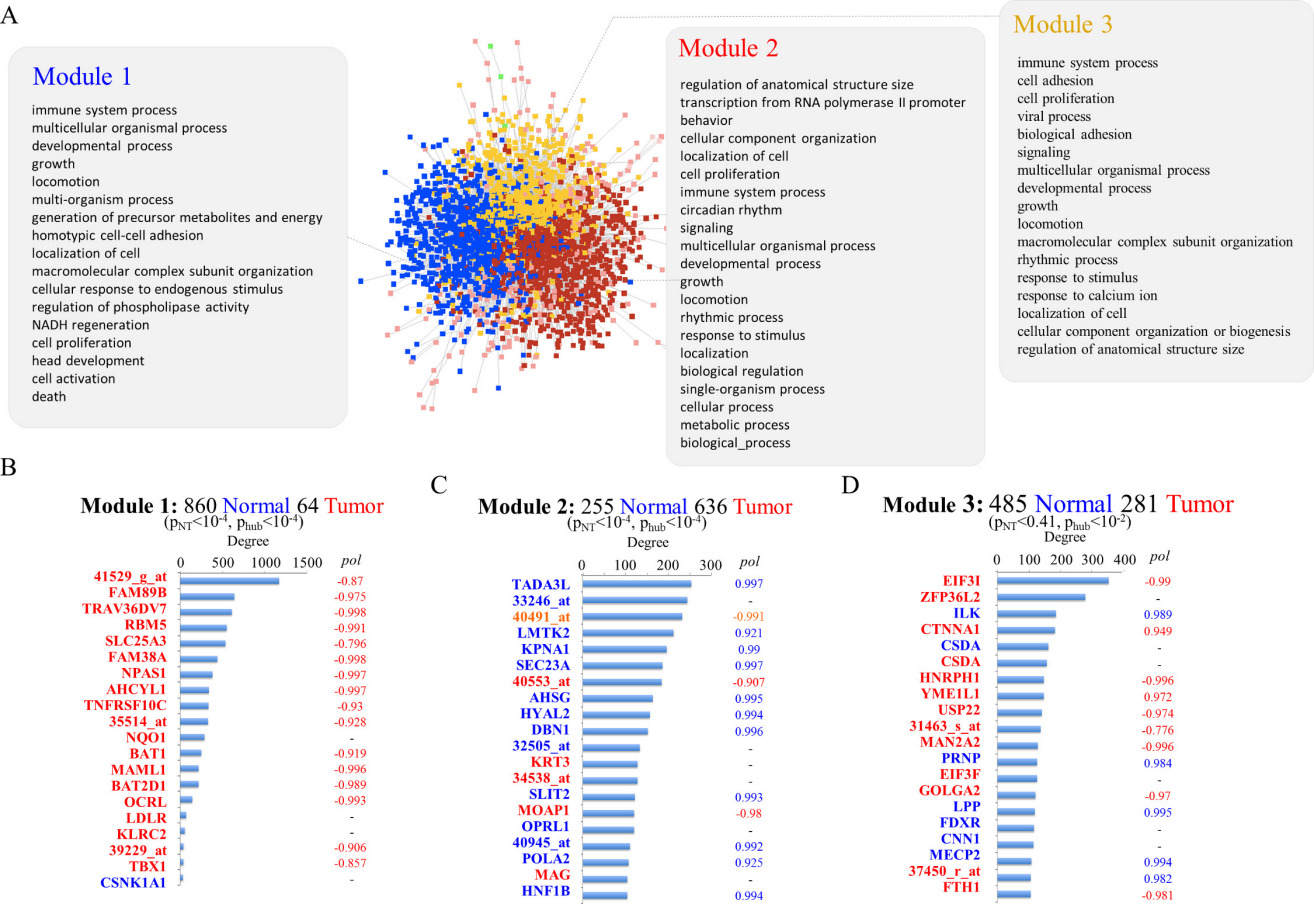
Co-expression network linking normal and tumour epithelial cells. The figures represent the modularized NT network with the results of the network connectivity and functional enrichment analysis. (A) The NT network in which genes belonging to the three main modules have been color-coded. The main functional terms identified by the web-based tool G:Profiler are listed in the three panels connected to each module. (B-D) The number of connections of the top 20 most connected hubs in each module. The x axis represents the number of connections, genes are represented on the y axis and color-coded to represent the cell type where they are expressed (blue and red represent normal and tumour epithelial cells respectively). Below each heading two p values are listed. p_NT_ is the p value from a test showing the probability that the proportion of normal/tumour expressed genes in each module is the result of random chance. p_hub_ is the p value from the test showing the probability that the proportion of normal/tumour expressed genes in the top 20 hubs is the result of random chance.

With this in mind, we first applied relevance networks [11], a relatively simple network inference procedure, to link genes differentially expressed in normal and in tumour epithelia. We used a dataset developed by Singh *et al*. [12], representing the transcriptional state of 47-paired prostate tumour and adjacent normal cells samples. The resulting network (NT network) is composed of 2581 positively and negatively correlated genes (**Fig 2**). These were subdivided in 1600 gene expression profiles in normal epithelia (referred from now on as ‘*normal-expressed*’ genes) and 981 gene expression profiles in tumour epithelia (referred from now on as ‘*tumour-expressed genes*’. The NT network was grouped into 68 modules by using GLay [13], a community detection method that maximizes inter-module connectivity. Only three modules contained more than twenty nodes and thus were selected for further investigation (**Fig 2**). This arbitrary threshold was used to make sure that a sufficiently large number of genes was present in each module for subsequent functional analysis. The NT network and its modules fitted a power law node connectivity distribution (p<10^−2^), consistent with the existence of a relatively small number of genes with a very large number of connections.

Module 1 displayed a marked enrichment in normal-expressed genes (**Fig 2B**, p<10^−4^) and module 2 showed enrichment in tumour-expressed genes (**Fig 2C**, p<10^−4^). In module 3, the frequency of normal- and tumour- expressed genes was as expected by random chance (**Fig 2D**, p = 0.41). Interestingly, the most connected genes in module 1 and 2 represented profiles from the tissue that was less represented (p<10^−4^). The most extreme case was module 1 where 19 of the 20 most connected genes were tumour-expressed genes (expected frequency was 1). Although module 3 showed no preferential tissue distribution, it still showed a higher than expected frequency of tumour-expressed genes among the 20 most connected genes (p<10^−2^). Functional analysis of the genes represented in each module showed that these were enriched in a wide spectrum of biological functions (**Fig 2A** and **S1 Table**).

### A novel topological index identifies putative directional signals linking normal and tumour epithelial cells

The results described above (**Fig 2**) are consistent with the notion that a relatively small number of genes expressed in either normal or tumour epithelial cells may control communication signals that can either modify or respond to the molecular state of the adjacent tissue.

In order to mine the NT network for such signals we developed the polarization index (*pol*), a novel gene connectivity metric. We design this index to represent genes that may exert an effect on the adjacent cell type only when expressed in one specific tissue. This scenario implies a directional signal, which is for example typical of soluble factors encoded by tumour suppressor genes or oncogenes. In the case of tumour suppressor genes, these may have lost the ability to control tumour cell proliferation via autocrine signaling but they may retain that function when expressed in adjacent stromal cells by a paracrine signal.

We formalized this scenario as follows:

Considering that a given gene *g*_*i*_ can be expressed in both normal and tumour tissue, we define *f*_*i*_ as the number of tumour-expressed genes that correlate with the normal-expressed *g*_*i*_. Similarly, we define *b*_*i*_ as the number of normal-expressed genes that correlate with the tumour-expressed *g*_*i*_. We define the polarization coefficient for gene *g*_*i*_ as:

$${pol}_{i}=\frac{f_{i}-b_{i}}{f_{i}+b_{i}+\varepsilon }$$ (1)

*ε* is a small positive constant designed to stabilize the ratio when *f*_*i*_ and *b*_*i*_ are small.

*Pol*_*i*_ has a number of desirable properties: its value is proportional to the asymmetry in the number of correlated genes with *gene i* in the two tissues while its sign gives the direction of the effect. This metric tend to 1 or -1 for f_i_ >>b_i_ or f_i_ << b_i_, respectively.

**Fig 3:**
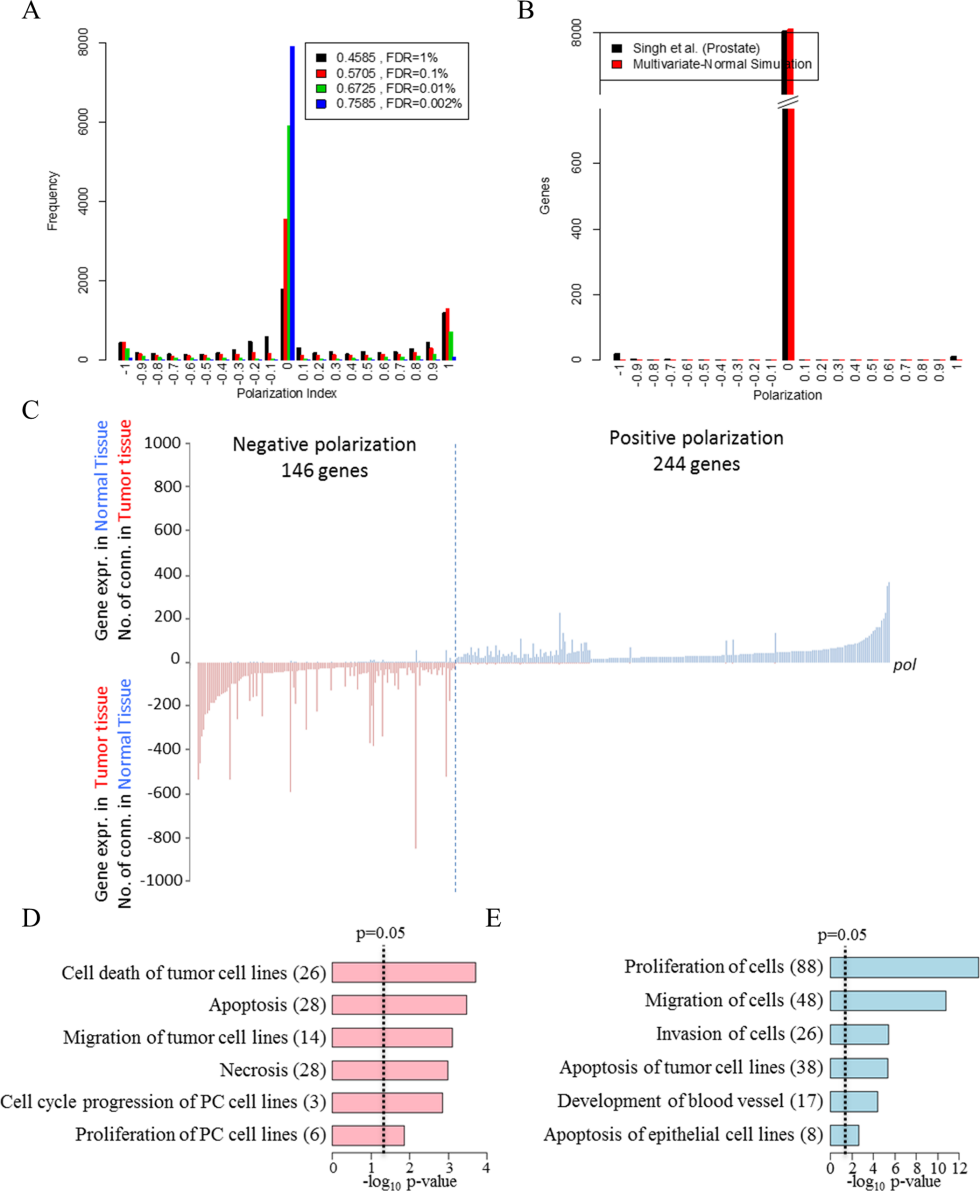
Analysis of the NT network using a novel topological index. The results of the analysis of the NT network using the polarization index. (A) The distribution of polarization coefficient for 4 different significance thresholds defying the NT network. (B) The same distribution from a multi-variate normal model that generates random datasets with similar distributions of correlation coefficients for the normal and tumour tissue but no constrains on the correlation structure between the two tissues. (C) The number of connections towards normal or tumour expressed genes for each of the selected (r_s_>|0.75|) polarized genes. The x-axis represent the polarized genes sorted by increasing values of polarization index. The y-axis shows the number of tumour-expressed genes correlated to each polarized gene when expressed in normal tissue (positive values) and the number of normal expressed genes correlated to each polarized gene when expressed in tumour tissue (negative values). (D-E) The number of genes and significance (x axis) for the most enriched functional terms in the negative and positively polarized genes respectively.

We computed this index for all genes represented in the NT network (**Fig 2**) and discovered that, independently of the threshold used, it is distributed accordingly to a multimodal distribution with three peaks (**Fig 3A**). The highest frequency of the distribution is centered on zero whereas a smaller number of genes show polarization coefficients close to +1 and -1.

We focused subsequent analysis on genes with pol>|0.75|, a very stringent threshold that we found to have less than 1 in 8000 false positives (**Fig 3B** and **S2 Fig**). This very stringent threshold identified 146 and 244 positively and negatively polarized genes, respectively (**Fig 3C** and **S2 Table**).

Functional analysis of the polarized genes using Gene Ontology and the Ingenuity database shows a statistically significant enrichment in functions key to cancer biology (**Fig 3D and 3E)**. The main functions significantly enriched in the negatively polarized genes are *cell death of tumour cell lines*, *migration of tumour cell lines*, *necrosis and proliferation of PC cell lines* (**Fig 3D**). The main functions enriched in the positively polarized genes are *proliferation of cells*, *migration of cells*, *invasion of cells and apoptosis of tumour cell lines* (**Fig 3E**). Moreover, 107 positively polarized genes (53% of the 204 genes that had functional annotation) are linked to the Gene Ontology term *cell communication* and therefore represent a class of proteins potentially mechanistically involved with cell crosstalk (**S3 Table**). Interestingly, only positively polarized genes are significantly enriched in this functional term (FDR<10^−2^). Manual curation into the role of the positive and negatively polarized genes using available literature and online databases was consistent with the computational analysis. In **Table 1** we report the positively and negatively polarized genes that are either secreted factors (potential paracrine signals) or factors partitioned at the cell surface (potentially involved in cell-cell communication via direct contact) or transcription factors that may regulate the expression of cell communication genes.

**Table 1:** Roles of the highly polarised genes.

**Positive Polarisation > 0.75**		
**Secreted**	Cell Migration	SLIT2
	Extracellular Matrix	HYAL2, LTBP1, COL16A1, DMBT1, SFTPD, FBLN1, MATN2, COL4A2, CBLN1, COL19A1
	Growth Factors / Other Secreted Factors	AHSG, DEFB4, DEFA3, PRB4, SVEP1, PRB4, TSHB, TGFB1
**Cell Surface**	Cell Adhesion / Focal Adhesion	MLLT4, AOC3, CEACAM3, CTNNA1, ADAM15, LPP, ILK
	Cell-Cell Communication	GJA1, GPA33, RAP1B
	Receptor	GEM, GABRG2, FGFR1, SIRPB1, GPR6, AVPR1B, IL9R, RCP9, PLXNA3, NCR3, GRIK5, TBXA2R, TNFRSF25, HTR4
**Regulatory**	Transcription Factors	TADA3L, HNF1B, TCF7, EPAS1, HOXD13, WWTR1, TP53, POU2F2, GATA2, HSF4, MYOG, PAX9, NEUROD2
**Negative Polarisation < -0.75**		
**Secreted**	Secreted	IGFBP5, WNT11, IGFBP2, IGFBP5, FGF9
**Cell Surface**	Cell-Cell Communication	NRXN1, LAMP2
	Receptor	IL1RL1, PTH2R, FGFR1, TNFRSF10C
**Regulatory**	TF	ETV3, BHLHB2, TBX1, PNN, MTA1, TBX19, BRD2, PAX7, CTBP2, ETV3, SIX3, USF2, MAML1

Additionally, almost all the network hubs described in **Fig 2** are characterized by a high polarization coefficient (either positive or negative).

### Predicted targets of polarized genes in tumour and normal epithelial cells represent a set of important cancer effector functions

In order to investigate the potential role of polarised genes in cell-to-cell communication we first identified their first neighbours in the NT network and then we tested the resulting gene lists for functional enrichment. We could identify 1223 normal-expressed genes as targets of tumour-expressed negatively polarised genes and 794 tumour-expressed genes as targets of normal-expressed positively polarised genes (**S4 Table**). We discovered that there was a significant overlap between them (520 genes, p<10^−3^) (**S3 Fig**) suggesting that although positively and negatively polarised genes are by definition different, they may ultimately target the same biological processes, in tumour and normal cells respectively. This hypothesis was supported by the functional analysis, which identified a set of terms enriched in the overlapping set of gene targets. Among these there were *regulation of cell death*, *response to growth factor*, *cell adhesion* and *extracellular region part* (**S3 Fig** and **S5 Table**).

### An *in vitro* co-culture system recapitulates the transcriptional changes predicted by the gene polarization analysis

We reasoned that if the cell-to-cell communication model we developed around the gene polarization index is correct, we should be able to modulate the putative targets of polarized genes by reconstructing an *in vitro* system where normal and tumour prostate cells share the same micro-environment. We performed such experiment by using a trans-well co-culture system where normal (RWPE1) and tumour (DU-145) epithelial cell lines are separated by a semipermeable membrane. In these experiments either tumour or normal cells were inserted into dishes already containing tumour cells. This experimental set up represents the aspect of the prostate tissue in which cancer epithelial cells sits in proximity but not necessarily are in direct contact (paracrine signals). Four sets of samples were processed for expression profiling 24 hours after the start of the experiment. These were: 1) RWPE1 cultured with RWPE1, 2) DU-145 cultured with DU-145, 3) RWPE1 cultured in the presence of DU-145 and 4) DU145 cultured in the presence of RWPE1. Genes whose expression in tumour cells is influenced by the presence of normal cells were identified by direct comparison between gene expression in DU-145 cultured on their own and gene expression in DU-145 cultured in the trans-well system in the presence of RWPE1. Similarly, we identified genes whose expression in normal cells depended on the presence of tumour cells by direct comparison between gene expression in RWPE1 cultured on their own and RWPE1 grown in the trans-well system in the presence of DU145. We considered the two sets of genes identified by this simple differential expression analysis as the experimental equivalents of the predicted targets of positively and negatively polarized genes, respectively.

**Fig 4:**
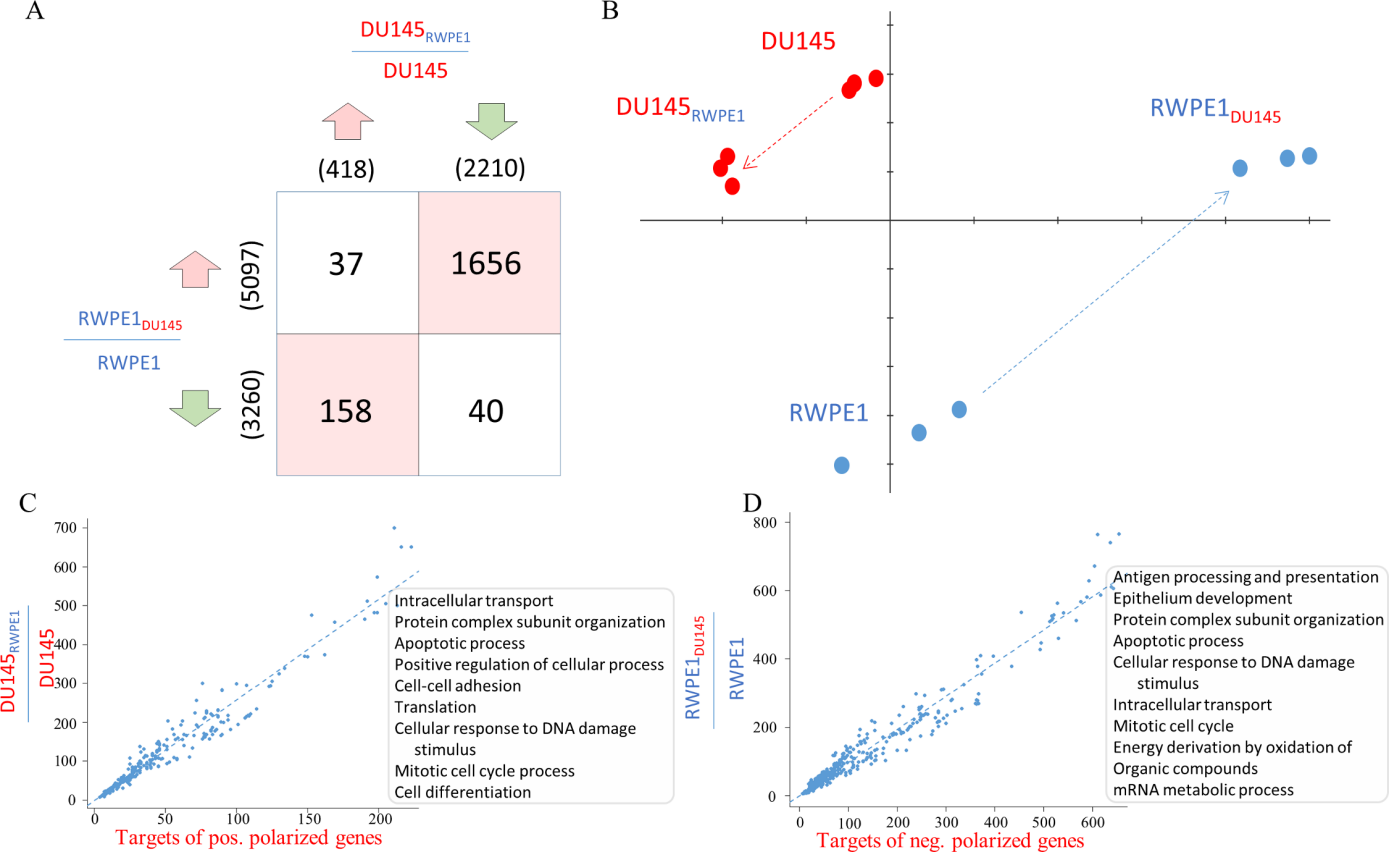
In vitro normal and tumour cell co-culture model. The results of the in vitro cell co-culture experiment used to analyze normal and tumour epithelial cells crosstalk. (A) Table showing the number of overlapping genes between differentially regulated gene lists in normal and tumour cells as a result of co-culture. Rows in the table represent up and down regulated genes in normal cells whereas columns are up and down regulated genes in tumour cells. There is a significant overlap in gene lists changing in opposite directions (p<0.0001, red background). (B) PCA plot representing normal and tumour cells cultured on their own (RWPE1 and DU145) or in co-culture (RWPE1_DU145_ and DU145_RWPE1_). (C-D) Scatterplots comparing the number of genes in functional terms represented in the predicted targets of polarized genes and genes differentially expressed in the co-culture model. Panel C represent tumour cells whereas panel D represent normal cells.

Consistent with the analysis of the targets of polarized genes (**S3 Fig**) we found a significant overlap between genes differentially expressed in normal and tumour cells as a result of co-culture (**Fig 4A**, p<0.01). We also discovered that a significant percentage of genes up regulated in tumour cells were down regulated in normal cells and vice versa (**Fig 4A**). This is consistent with the results of a principal component analysis of these data showing that the variation between normal and tumour cells following co-culture followed anti-parallel trajectories (**Fig 4B**).

Next, we compared the predicted targets of polarized genes and the experimentally determined transcriptional signatures. The overlap between the differentially expressed genes in the co-culture system and the predicted targets of polarized genes was significant both at gene (**S4 Fig**) and at functional level (**Fig 4C and 4D**). We concluded that remarkably, the *in vitro* system was able to recapitulate a significant component of the transcriptional network inferred from the clinical study.

**Fig 5:**
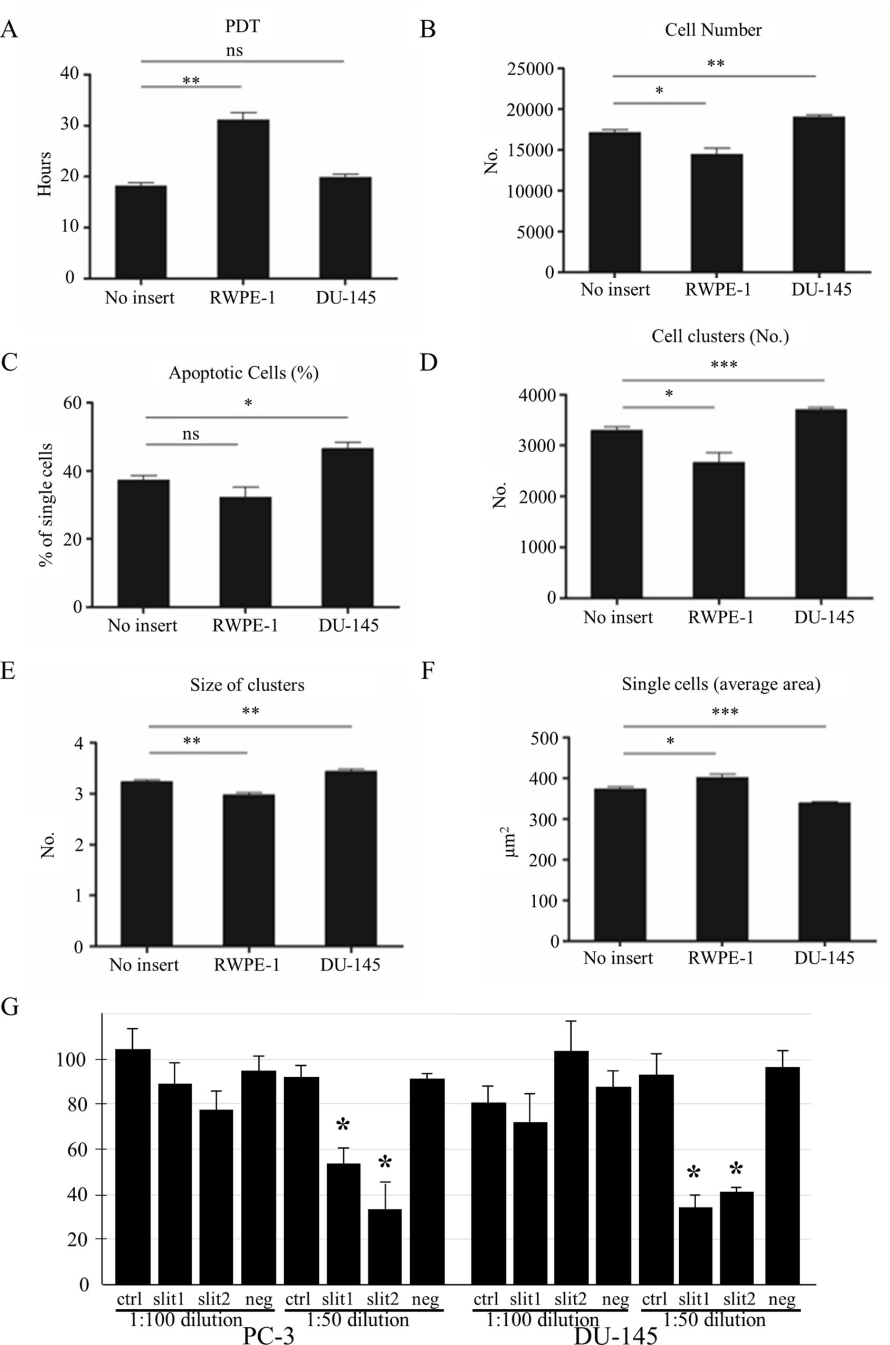
Phenotypic analyses of tumour cells in co-culture experiments. In each panel is shown the phenotypic characteristic of the tumour cells alone (DU145, no insert), the tumour cells in the presence of normal (RWPE1) or tumour cells (DU145). The presence of normal cells are seen to (A) increase the population doubling time (PDT), (B) decrease the total cell number, (C) have no significant effect on apoptosis, (D) decrease the number and (E) size of the cell clusters and (F) increase the number of single cells. Each of these changes shows a normalisation of the phenotypic characteristics of the tumour cells by the presence of the normal cells. In contrast co-culture with DU145 tumour cells is seen to have the opposite effect and to increase the tumour phenotype of the tumour cells. Panel G shows the results of a clonogenic assay performed on two different tumour cell lines (DU145 and PC-1). The figure shows that the addition of 1:50 dilution of culture media, conditioned by over-expressing Slit-2 induce at least 60% reduction in cell survival respect to control cultures.

The functional analysis of these gene signatures revealed enrichment in several important cellular functions that are very relevant in cancer (e.g. *regulation of growth*, *apoptosis* and *cell adhesion*). Since we could not identify a specific direction in differential gene expression we set to determine whether change in the transcriptional state of co-cultured cells impact a relevant cancer phenotype. We therefore performed a battery of *in vitro* tests on tumour cells, using the same trans-well co-culture system described above. Here we assessed whether the transcriptional signatures defined by our computational analysis and validated by the *in vitro* co-culture system may truly reflect a cancer relevant phenotype. We found that the presence of normal epithelial cells induced several phenotypic changes in tumour cells. More specifically, population doubling time (PDT) in tumour cells cultured in the presence of normal cells was considerably longer than in tumour cells cultured on their own (30 hours against 18 hours, **Fig 5A**). Cell numbers at the end of the experiment were consistent with this finding and also revealed that additional tumour cells in the trans-well promoted survival (**Fig 5B**). The apoptosis test revealed that normal cells did not have any effect but tumour cells surprisingly increased the number of tumour apoptotic cells (**Fig 5C**). We then tested the formation of cell clusters and recorded the number of cell clusters (**Fig 5D**), the size of clusters (**Fig 5E**) and the area of the dish occupied by single cells (**Fig 5F**). Normal cells reduced the number and size of clusters and increased the area occupied by single cells whereas tumour cells had the opposite effect (**Fig 5D–5F**).

Consistent with these findings, conditioned media from COS cells overexpressing the tumour suppressor gene SLIT2, one of the most positively polarized genes (pol = 0.99) which is expressed at higher levels in normal prostate tissue compared to tumour (**S5A Fig**), was able to dramatically reduce tumour cell clone formation in a Matrigel in vitro Clonogenic assay (**Fig 5G**).

All of this data is consistent with the normal cells effectively 'normalising' the phenotypic characteristics of the tumour cells.

### Genes with a high polarization index and linked to the genotype of tumour cells are predictive of clinical outcome and are over-expressed in normal prostate tissue

Having inferred and experimentally validated a transcriptional network representing the interaction between normal and tumour prostate epithelial cells we then hypothesised that expression of genes within the network may be influenced by genetic/epigenetic modifications and/or correlate to tumour features and clinical outcome.

**Fig 6:**
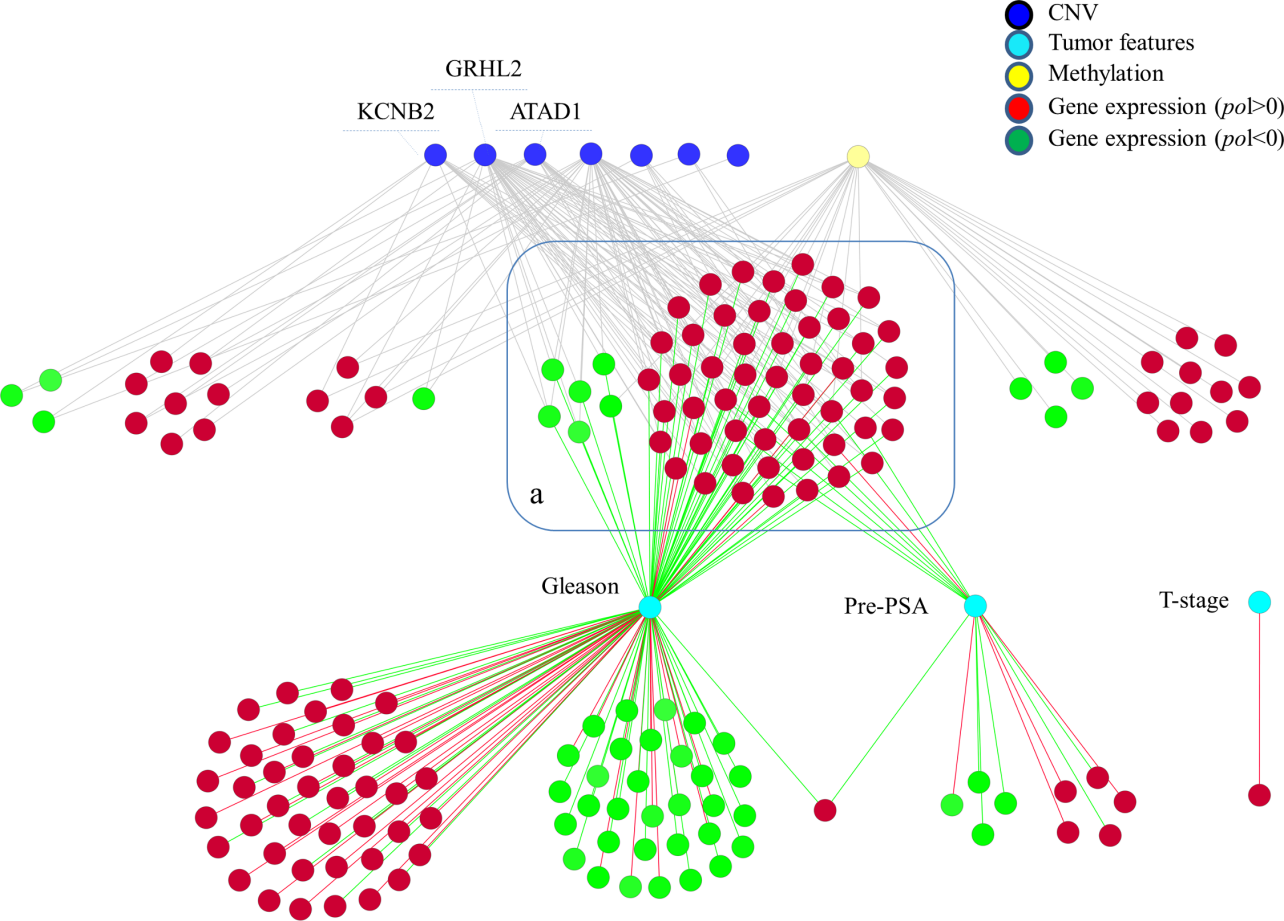
Integration of CNV, polarized genes, mRNA expression and tumour features. The figure summaries the relationship between gene CNV (blue), mRNA re-expression following hypo-methylating agents (yellow), polarized gene expression (red and green nodes are positively and negatively polarized genes, respectively) and tumour features (cyan). These relationships are the result of the statistical modeling described in the last section of the Results. Genes within the blue open rectangle (a) are under the influence of CNV and are linked to tumour features. Table 1 lists the genes within panel a and their functional classification according to Gene Ontology.

We first checked whether the expression of polarised genes might be influenced by DNA methylation, a common mechanism for transcriptional silencing in cancer. By mapping genes known to be re-expressed in prostate cancer cell lines, following exposure with DNA hypomethylating agents [14][15][16], we could show that methylation significantly affect the expression of 30 of the 245 positively polarized genes and 12 of the 146 negatively polarised genes in tumour cells (**S6 Fig and Fig 6** and **S6 Table**). Although the percentage of genes affected by methylation is relatively small, the number of positively polarised genes whose expression is affected by methylation was significantly higher than expected by random chance (**S6 Fig**).

Next we assessed the role of copy number variation (CNV). We selected an independent dataset [17], which included genetics (CGH), gene expression and clinically relevant variables (**S7 Table**). First we tested whether the expression of polarised genes was directly affected by CNV. We could only identify 9 polarised genes with significant correlation (p<0.01) between their CNV and expression (**S8 Table**). Next we developed a hierarchical Bayesian model to identify whether epistatic CNV could explain the expression of polarized genes in tumour cells. We were able to show that the expression of 70 polarised genes could be explained by CNV in seven genomic regions (**S9 Table** and **Fig 6**). Three of these included genes with known function (*ATAD1*, *GRHL2* and *KCNB2*) (**Fig 6** and **S10 Table**). Interestingly, the large majority of polarized genes whose expression was linked to CNV were mainly positively polarised (59 out of 70).

Finally, we tested whether the expression of polarised genes was related to tumour features and clinical outcome. Indeed we found that the expression of a large number of polarised genes (130) was linked to Gleason score. A smaller number of genes (18 and 1) were linked to PSA antigen and T stage, respectively (**Fig 6** and **S11 Table**).

The integration of these associations using a network representation revealed 173 polarised genes linked either to regions affected by CNV (89 genes) and/or to tumour features (84 genes) (**Fig 6**). Remarkably, while the expression of none of the polarised genes could be linked to survival, 132 of them were linked to time free of recurrence (FDR<5%, **S7A Fig** and **S12 Table**). Interestingly we could also show that polarised genes linked to CNV did show significantly lower p-values than polarised genes only linked to Gleason score (**S7B Fig**) supporting the clinical relevance of the epistatic effects identified by the computational model.

A group of 58 positively polarised genes and 6 negatively polarised genes (**Fig 6 and S7B Fig** and **Table 2**) were linked to both CNV and Gleason score. We found that the large majority of genes in this group (57/58) were negatively associated to Gleason score and positively correlated to time free of recurrence (**S7B Fig** and **S12 Table**).

**Table 2:** Functional profile of genes linked to CNV and Gleason score.

Gene Symbol	Description	Polarization	Cellular Component	Biological Process
SEC23A	Sec23 homolog A (S. cerevisiae)	1.00	Golgi membrane	Intracellular Protein Transport
DBN1	Drebrin 1	1.00	Cytoskeleton	Cytoskeleton organisation
LPP	LIM domain containing preferred translocation partner in lipoma	0.99	Plasma membrane	Cell adhesion
CNN1	Calponin 1	0.99	Cytoskeleton	Cytoskeleton organisation
SLIT2	Slit homolog 2	0.99	Extracellular region	Cell morphogenesis
ILK	Integrin-linked kinase	0.99	Cytoskeleton	Cell morphogenesis
FAM114A1	Family with sequence similarity 114, member A1	0.99		
MYL9	Myosin, light chain 9, regulatory	0.99	Cytoskeleton	regulation of muscle contraction
CCND2	Cyclin D2	0.99	Cyclin-dependent protein kinase holoenzyme complex	egulation of cyclin-dependent protein kinase activity,
PRNP	Prion protein	0.98	Endoplasmic reticulum	protein complex assembly
LTBP1	Latent transforming growth factor beta binding protein 1	0.98	Extracellular region	KEGG_TGF beta signalling pathway
MAOB	Monoamine oxidase B	0.98	Mitochondrion	oxidation reduction,
DKFZP564O0823		0.98		
PALLD	Palladin, cytoskeletal associated protein	0.98	Cytoskeleton	Cytoskeleton organization
WWTR1	WW domain containing transcription regulator 1	0.98	Nucleoplasm	Negative regulation of transcription from RNA polymerase II promoter
AGPS	Alkylglycerone phosphate synthase	0.98	Mitochondrion	lipid biosynthetic process,
CLIC4	Chloride intracellular channel 4	0.98	Cytoskeleton, Plasma membrane	Ion transport
CALD1	Caldesmon 1	0.98	Cytoskeleton, Plasma membrane	Cell motion
DYRK2	Dual-specificity tyrosine-(Y)-phosphorylation regulated kinase 2	0.98		Regulation of glycogen biosynthetic process
ITPR1	Inositol 1,4,5-triphosphate receptor, type 1	0.98	Membrane Fraction	Calcium ion transport
DPYSL3	Dihydropyrimidinase-like 3	0.98	Cytoskeleton	KEY_Cell projection
FLNC	Filamin C	0.97	Cytoskeleton	KEGG_ Focal adhesion
FHL1	Four and a half LIM domains 1	0.97	Cytosol	Regulation of cell size
DIDO1	Death inducer-obliterator 1	0.97	Cytoskeleton	Transcription
PDE4D	Phosphodiesterase 4D, cAMP-specific	0.97	Cytoskeleton	Purine nucleotide metabolic process
KIFC1	Kinesin family member C1	0.97	Cytoskeleton	Mitotic sister chromatid segregation
ZFP36	Zinc finger protein 36	0.97	Cytosol	Nuclear-transcribed mRNA catabolic process
NUCB1	Nucleobindin 1	0.97	Cytoskeleton, Extracellular region	
MTHFD2	Methylenetetrahydrofolate dehydrogenase	0.97	Mitochondrion	One-carbon metabolic process
FOS	v-fos FBJ murine osteosarcoma viral oncogene homolog	0.97	Nucleoplasm	Response to reactive oxygen species
FBLN1	Fibulin 1	0.96	Extracellular region	
CRYAB	Crystallin, alpha B	0.96	Cytoskeleton	Microtubule cytoskeleton organization
FLNA	Filamin A, alpha	0.96	Cytoskeleton	Cytoskeleton organization
PFKP	Phosphofructokinase, platelet	0.96	Cytosol	Monosaccharide metabolic process
RBPMS	RNA binding protein with multiple splicing	0.96		RNA processing
RAB3GAP1	RAB3 GTPase activating protein subunit 1	0.96	Soluble fraction	Regulation of GTPase activity
WDR1	WD repeat domain 1	0.96	Cytoskeleton, Extracellular region	Sensory perception
C9orf61		0.96		
TPM2	Tropomyosin 2 (beta)	0.95	Cytoskeleton	Regulation of ATPase activity
DES	Desmin	0.95	Cytoskeleton	Cytoskeleton organization
PRB4	Proline-rich protein BstNI subfamily 1	0.95	Extracellular region	
MAP1LC3B	Microtubule-associated protein 1 light chain 3 beta	0.95	Cytoskeleton, Vacuole	Proteolysis
MATN2	Matrilin 2	0.95	Extracellular region	
MBNL1	Muscleblind-like (Drosophila)	0.94	Cytoskeleton	Spliceosome assembly
DDX3X	DEAD (Asp-Glu-Ala-Asp) box polypeptide 3, X-linked	0.91	Nucleoplasm	
CAP1	CAP, adenylate cyclase-associated protein 1 (yeast)	0.89	Cytoskeleton	Cell morphogenesis
KRTAP26-1	Keratin associated protein 26–1	0.88	Cytoskeleton	
PAFAH1B1	Platelet-Activating Factor Acetylhydrolase 1b, Regulatory Subunit 1 (45kDa)	0.86	Cytoskeleton	
SPOP	Platelet-activating factor acetylhydrolase, isoform Ib, subunit 1 (45kDa)	0.80	Astral microtubule	M phase of mitotic cell cycle
MYH11	Myosin, heavy chain 11, smooth muscle	0.80	Cytoskeleton	Cytoskeleton organization
ZMYND11	Zinc finger, MYND domain containing 11	0.77		Regulation of transcription
MTX1	Metaxin 1	0.75	Mitochondrion	Mitochondrial transport
SORBS2	Sorbin and SH3 domain containing 2	-0.80	Cytoskeleton	
ACTN1	Actinin, alpha 1	-0.90	Cytoskeleton	Cytoskeleton organization
RTN4	Reticulon 4	-0.92	Nuclear envelope	Angiogenesis
BAT1	HLA-B associated transcript 1	-0.92	Nucleoplasm	RNA splicing
PDE8A	Phosphodiesterase 8A	-0.99		Purine metabolic process
RBM5	RNA binding motif protein 5	-0.99	Nucleolus	Spliceosome assembly

Intriguingly, these were highly enriched in Cytoskeleton proteins (24 out of 51, over-represented in the GO term *Cytoskeleton* at FDR<10^−8^) (**Table 2**).

**Fig 7:**
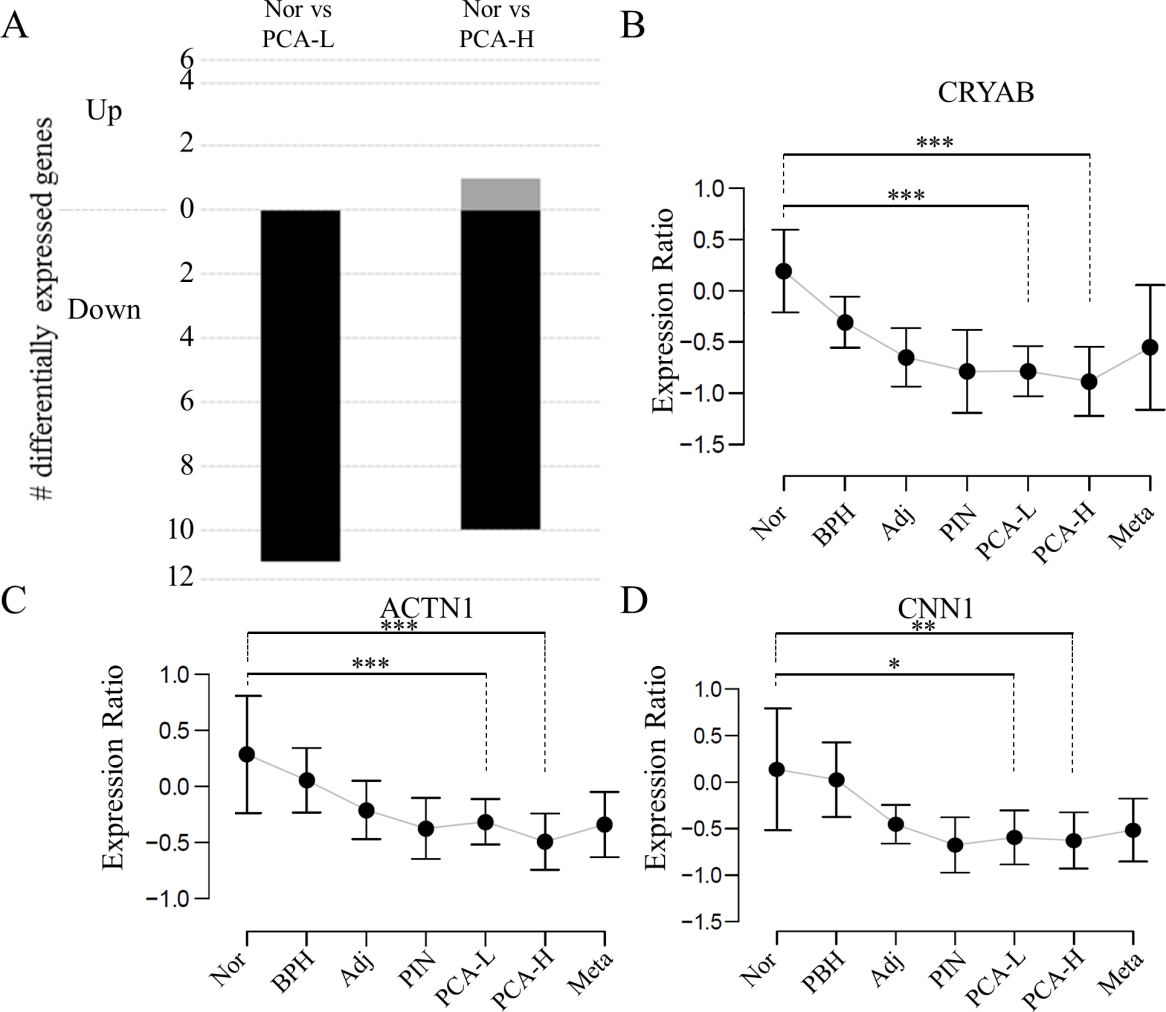
Expression of polarized genes in laser-capture micro-dissected tumour and normal tissue. The differential expression patterns of polarised genes linked to CNV and Gleason score in the Tomlins *et al* LCM-based gene expression dataset. 36/58 genes were mapped from the network (see Fig 5) to the independent dataset. (A) Expression of all but 1 of the polarised differentially expressed in the LCM dataset (11/36) was downregulated in low and high-grade tumour tissue compared to healthy prostate. (B-D) Expression of several polarised genes linked to CNV and Gleason score in specific prostate tumour compartments isolated by the Tomlins *et al* study. Nor = normal prostate tissue, BPH = benign prostate hyperplasia, Adj = prostate tissue adjacent to tumour, PIN = intraepithelial neoplasia, PCA-L = low-grade tumour, PCA-H = high-grade tumour, Meta = metastatic tumour tissue. * p < 0.05, ** p < 0.01 *** p < 0.001.

We then tested the expression of 36 out of the 58 genes that were profiled in a dataset representing normal and tumour cells which were laser micro-dissected from prostate cancer specimens [18] (**Fig 7**). This analysis showed that 11 out of 36 are differentially regulated and that all except 1 were down regulated in the tumour tissue (**Fig 7**), an observation that is consistent with the direction of correlation with the survival free of metastases.

Among these 58 genes, 8 represented genes involved in formation of cell projections (ACTN1, CALD1, CLIC4, DPYSL3, DBN1, ILK, PAFAH1B1 and RTN4) and six (ACTN1, CCND2, FLNA, FLNC, ILK and MYL9) mapped on the KEGG pathway *focal adhesion* (FDR<1%). Also, five were proteins known to be associated to the Golgi apparatus and involved in protein secretion (SEC23A, CRYAB, FLNA, NUCB1 and PRNP). Among these were several genes with known tumour suppressor activity (e.g. FLNA [19], FBLN1 [20], MYL9 [21], CLIC4 [22] and SEC23A[23]).

## Discussion

Here we have described a relatively simple network inference and analysis procedure, explicitly designed to learn cell communication networks from observational data. This is the first example of an open ended reverse engineering strategy that explicitly searches for cell communication networks from observational data. Our approach also provides clues on the role of normal epithelial cells in prostate tumour progression. The application of our analysis strategy (**Fig 1**) to prostate cancer revealed that normal epithelial cells may have a more important role in controlling tumour expansion than previously suspected. The applicability of this approach is broader and indeed it opens important avenues for better understanding the whole network of signals regulating cell communication in both normal and pathological scenarios.

### The role of normal epithelial cells in tumour progression

Since the large majority of efforts have focused on understanding the role of fibroblast and endothelial cells in cancer, the interface between normal and transformed epithelial cells is still not clearly understood. Our analysis suggests that normal epithelial cells exert a “normalizing” effect on tumour cells, up to an advanced stage of tumour progression.

A number of recent studies have suggested that at the initial phase of tumour expansion, normal epithelia could provide a tumour suppressive environment that cancer cells need to overcome to develop a tumour. So far, tumour suppressor activity of normal epithelial cells has been studied in cell culture systems replicating early transformation events in epithelia [24]. These models include kidney and mammary epithelial cells in culture where only a few cells are selectively transformed by oncogenic transformation or inhibition of tumour suppressor genes [24]. In these conditions, transformed cells are excluded from the epithelia and out grown by normal epithelial cells. It has been suggested that additional mutations and/or alterations in the adhesion properties of tumour cells may be needed to overcome the tumour suppressive effects and allow for clonal expansion [24]. However, the precise molecular events underlying this process are still unknown.

Our work therefore provides further evidence of the tumour suppressor effects of normal epithelial cells and supports the concept that although tumour cells obviously eventually overcome these normalizing signals, the effect of normal epithelia may be relevant for the entire clinical history of prostate cancer.

### Potential mechanisms mediating the anti-tumour activity of normal epithelial cells

The models we have developed provide a link between genetic mutations and the expression of polarized genes in tumour cells. Remarkably, the functional profile of mutated genes is consistent with a pivotal anti-tumour role of the apical junctional complex and the protein secretion machinery.

Among the three genes we have identified as potential epistatic regulators, GRHL2 is known to be a transcription factor known to play a pivotal role in cancer progression [25][26][27]. GRHL2 regulates epithelial cell differentiation by effectively regulating the expression of genes of the epithelial apical junctional complex [28]. It controls the expression of the adherents junction gene E-cadherin and the tight junction gene claudin 4 (Cldn4) and has been linked to both pro and anti-tumour activity [25]. Moreover, GRHL2 up regulates the human telomerase reverse transcriptase (hTERT) gene during cellular immortalization of oral squamous cell carcinoma cells [29]; it is a proto-oncogene in breast cancer cells [25]; it regulates proliferation of hepatocellular carcinoma cells [30] and is a suppressor of epithelial-to-mesenchymal transition in breast cancer [31].

Our model predicts that increase expression of GRHL2 due to CNV down regulates the expression of a set of polarized genes that precisely encode for components of the cytoskeleton and are involved in focal adhesion and cell migration. Several of these genes are extracellular factors and one of them (SEC23A) has been found to control secretion of anti-tumour factors in breast cancer [23].

These observations lead to the hypothesis that increased expression of GRHL2 in tumour cells may result in the deregulation of at least two different types of tumour suppressor signals, one dependent on the establishment of focal adhesion junctions and the other directly affecting secretion of anti-tumour factors. This chain of events may contribute to tumour transformation and metastases formation and at the same time could make tumour cells sensitive to the same tumour suppressor signals that continue to be produced by adjacent normal epithelial cells. The in vitro system we have used to validate our model shows that normal epithelial cells are able to exert anti-tumour effects even if normal and tumour epithelial cells were separated by a semi-permeable membrane, suggesting that soluble factors may be playing a major role in tumour suppression. Secretion of the highly positively polarized gene SLIT2 from normal epithelial cells has the potential of exerting a tumour suppressor activity as shown by our clonogenic assay on tumour cells exposed to diluted conditioned media.

More broadly, there is strong support in the literature linking several of the positively polarized genes to tumour suppression.

More precisely, FLNA[19] FBLN1[20], MYL9 [21], CLIC4 [22] all have demonstrated tumour suppressor activity. It has been shown that Filamin A (FLNA) exerts anti-tumour activity via at least three different mechanisms. It represses MMP-9 expression reducing cell migration in prostate cancer. It controls focal adhesion and androgen-related cell migration in human fibrosarcoma [19] and Cyclin D1/cyclin-dependent kinase 4 mediated cell migration in breast cancer [32]. The myosin light chain (MYL9) in stroma has been shown to predict malignant progression and recurrence-free survival in prostate cancer [21]. Fibulin 1 (FBLN1) is down regulated in a number of tumours, including prostate [33]. CLIC4 was first characterized as intracellular chloride channel, later shown to be involved in signaling, cytoskeleton integrity and differentiation [34] and is a tumour suppressor gene in cutaneous squamous cell cancer [22].

### Computational inference of cell-to-cell communication networks

The reverse engineering approach we have adopted is based on the assumption that gene co-expression is either directly or indirectly a reflection of important underlying mechanisms of gene regulation and as such it can reveal novel biological networks. While this concept is well accepted in the scientific community, it remains true that correlation does not necessarily imply causation, hence the importance of experimental validation.

However, for a number of candidates, it is possible to hypothesize a mechanism whereby highly polarized genes may directly affect adjacent cells. For example, a number of them are secreted factors that can work as paracrine signals or membrane proteins known to be involved in cell communication (**Table 1**). This is the case for Slit-2 that we have experimentally verified by treating prostate cancer cells with conditioned media derived from cells over-expressing the recombinant protein. Others may indirectly control cell communication. This is for example the case with transcription factors (e.g. GATA2 control of IGF1 signalling [35]), proteins controlling secretion (e.g. LTBP1 control of TGFB1 secretion [36]) or proteins involved in cell migration.

Interestingly, the gene expression profiling analysis we have performed to validate our predictions, suggest that the polarization coefficient may have the ability to capture directional signals that are triggered by normal and tumour cells. However, the experimental system we have used is based on a trans-well system, which only validate paracrine signals.

We believe our approach could have a broad impact. Although, at present there are not many suitable datasets containing both disease and adjacent normal tissue, we have verified that the distribution of polarization coefficient in two additional, datasets representing kidney and liver adjacent normal and tumour tissues are similar to the one observed in prostate cancer **(S9 Fig**). In the future we envisage that tissue laser micro dissection and mRNA sequencing technologies may provide a very powerful combination for the identification of genome wide cell communication networks.

### Conclusions and further developments

The approach we have developed has the advantage to reverse engineer cell communication networks in the absence of any prior information. In this respect, the method is different from the recently developed computational method developed by Choi *et al* [10]. The latter has been successfully applied to understanding the relationship between stroma and cancer cells in a model of lung tumour metastases and is based on comprehensive ligand-receptor network information, which can be extracted from several knowledge databases. We envisage that the integration of these knowledge driven approach within the framework of statistical learning will allow the development of a more powerful set of methodologies.

The study we have performed relies on cross-sectional data and therefore the correlations we estimate do not take into consideration the hierarchical sequence of events that characterize cell communication dynamically. However, such dynamics can be captured using *in vivo* models of tumor expansion [37]. In such scenarios, different computational methodologies may be used to reverse engineer underlying gene regulatory networks [38]. For example, suitable approaches may include ordinary differential equation (ODE) or state space models [39].

In conclusion, the approach we have pioneered is likely to provide a general strategy to ‘*learn*’ the structure of cell-to-cell communication networks in diseased and physiologically normal tissues. We anticipate that the availability of a viable strategy to infer cell communication networks will stimulate the development of experimental studies representing the molecular state of adjacent tissues and their functional interactions in physiology and disease.

## Methods

### Microarray datasets

This analysis initially focus on the dataset developed by Singh et al.[12] representing the transcriptional state of 47 paired prostate tumour and adjacent normal cells samples. Raw Affymetrix microarray data were normalized and processed before analysis to remove low variant and low expressed genes. Further details of the procedures can be found in **S1 File**.

The analysis linking copy number variation (CNV), gene expression, tumour features and clinical outcome was performed on the dataset developed by Taylor et al. [17], which consist of 231 tumour samples. Raw comparative genomic hybridization Agilent data was processed as detailed in **S1 File**.

The dataset developed by Tomlins *et al* [18] was used to test the expression of polarized genes in laser capture micro-dissected low and high-grade tumour and normal prostate tissue. In the Tomlins et al study, tumour grading was determined by Gleason score. A scores of 3 determined a low-grade tumour, a score of 4 or greater determined a high-grade tumour. Raw data was downloaded and normalized using the “marray” BioConductor package in R [40].

All data processing was performed in the statistical environment R.

### Network inference

Network inference was performed using a relevance network approach [11]. Non-linear Spearman ranking correlation (**r**_**s**_) was used to infer gene-to-gene correlations. In order to estimate the number of significant correlations, 100 bootstrap versions of the original dataset were used for each dataset to draw the null distribution of **r**_**s**_ expected by chance. The bootstrap distribution was used to estimate a p-value, which was subsequently corrected for multiple-test using an FDR correction procedure [41] (**S1 Fig**). The use of the relevance networks based on the Spearman correlation coefficient has advantages respect to more complex reverse engineering methods such as the mutual information based ARACNE [42] algorithm. Spearman correlation measure both positive and negative correlations and is better suited for datasets with a smaller number of samples. We used a threshold of **r**_**s**_ >|0.75| (FDR<10^−2^) to select significant connections (NT network).

### Network modularization and analysis

The NT network, representing statistically significant correlations between genes expressed in normal and tumour tissues, was modularized using the community finding algorithm GLay [13], as implemented in the network analysis tool Cytoscape [43].

The algorithm begins by setting each node into a separate community and progressively merges those with the maximum increase to the modularity score. The hierarchical merging tree is cut at the point where maximum modularity is achieved.

Connectivity analysis of the whole network and of the three largest modules (defined as larger than 20 nodes) was performed using the network analysis tool *NetworkAnalizer* [44], also implemented as a Cytoscape plug-in.

### The polarization index

The general definition of the *polarization index* for a given gene *i*, have been given in the result section (**Eq 1**). The analysis described in this paper has been performed with the parameter *ε* set to 1. Additionally, *pol* was set to 0 if the absolute difference between *f* and *b* was lower than 20 to avoid high *pol* values for low number of connections.

### Computational validation of the polarization index

In order to acquire confidence in the biological relevance of the polarization index we derived a null hypothesis distributions for estimating the likelihood that a given polarization value derives by random chance. This represented a scenario where the overall properties of the data are conserved in the absence of any interaction between normal and tumour samples.

Random data sampled from the Singh *et al*. dataset were used to compute normal and tumour correlation matrices. Each matrix was fitted by a multivariate Gaussian model to generate a synthetic dataset. Synthetic datasets were then used to compute the correlation matrix whose distribution predictively resembles that of the original dataset. Subsequently, the polarization index was estimated from this correlation matrix. The multivariate fitting and subsequent random dataset generation was performed using the function *rmvnorm* within *mvtnorm* packages [45] in the R statistical software environment (**S2 Fig**). Significantly polarized genes have been defined as *pol*_*i*_ > |0.75|. At this threshold we did not observed any false positives in the 8000 random simulations performed.

Although the expected level of contamination of tumour tissue with normal cells is expected to be very low, we devised a computational strategy to ask whether the polarization index could arise as a result of contamination of tumour samples with normal cells. We computed the polarization index between two simulated datasets that reproduce a situation where both tumour and normal samples are derived from normal tissues with added noise, thereby simulating variation that is consistent with a true microarray experiment (**S8A Fig**). Firstly, an adapted model of the type derived by Jain *et al* [46] was used to estimate the experimental noise across replicates (**S8B Fig**). Random Gaussian noise [47] derived from this noise model was then added to the normal tissue dataset to create a synthetic normal and synthetic contaminated tumour dataset. The intensity of the added noise was controlled by adding a scaling factor γ, which was chosen to match the distribution of correlations between genes in the synthetic datasets with the distribution observed in the real data (**S8C Fig**). The distribution of the polarization coefficient is consistent with the notion that even high levels of contamination cannot explain the observed distribution of polarization coefficient (**S8D Fig**).

### Polarization in kidney and liver expression profiling datasets

In order to test whether the trimodal distribution of the polarization coefficient could be observed in other cancer types in addition to prostate cancer, we analyzed two additional public domain datasets representing kidney [48] and liver [49], respectively. Only paired data corresponding to tumour and normal from the same tissue were used. Only one pair of samples per individual was used. In general, normal tissue was adjacent to the tumour. Datasets were normalized and processed before analysis as for the main prostate cancer dataset. Results are shown in **S9 Fig**.

### Functional analysis

Lists of polarized genes or their correlated genes were analyzed for enrichment of curated functional categories using the QIAGEN Ingenuity Pathway Analysis tool (IPA, www.qiagen.com/ingenuity). Enrichment of Gene Ontology (GO) terms and KEGG pathways was determined using the web-based tool gprofiler [50]. In order to reduce redundancy in the functional terms we used REVIGO and selected the functional terms with dispensability index equal to zero. Unless stated otherwise gProfiler functional clusters were considered for further investigation if they had a FDR<1%.

### Co-culture system

Normal (RWPE1) and tumour (DU145) prostate cell lines were co-cultured in a transwell system (transwell I used was from Nunc, Loughborough, UK, Cat. 12-565-286; Pore size, 0.2 μm.) for 24 hours in the presence of DMEM containing 10% fetal calf serum. The experiment was performed in triplicate with DU145 alone or DU145 co-cultured with either RWPE1 or DU145 in the insert. Cells from all compartments were harvested and RNA extracted using a Qiagen RNeasy kit according to the manufacturer's instructions (Qiagen, USA). Custom-made oligonucleotide arrays were manufactured using the Operon Human Oligo set, version 3.0 [51] and then hybridized with Cy3 labeled probe, as described in Sarti *et al*. [52]

Phenotypic cell analysis was carried out in Becton Dickinson TC treated 96-well plates. 2.5 x 103 cells were seeded per well in DMEM containing 10% fetal calf serum. 24 hours later, some wells were fitted with inserts also seeded with 2.5 x 103 cells per insert. Two days later inserts were removed, media was aspirated from the wells and cells were fixed with 85% ice-cold ethanol for at least two hours. After fixation cells were stained with propidium iodide (10 μg per ml propidium iodide, 100 μg per ml RNase A, 0.1% Triton X-100 in PBS, 100 μl per well). Plates were incubated at 37°C for 20 min in the dark and then analysed by laser scanning cytometry (Acumen Explorer, TTP Labtech.).

The intensity of the propidium iodide fluorescence was proportional to the DNA content of the cells and was measured on a linear scale. Single healthy and apoptotic cells were identified based on nuclear size and DNA content [53]. Cell clusters were defined as single scanned objects that contained multiple nuclei. The size of the clusters was defined as the ratio of the total nuclear area within a cluster divided by the size of an average nucleus in the same population.

### Clonogenic assay

Single-cell suspensions for either PC-3 or DU 145 cells, were prepared from 80% confluent cultures. The cells were counted and plated onto 24-well flat-bottomed plates using a two-layer soft agar system with 1x10^3^ cells in 400 μl of media per well, as described previously [54]. The feeder layer was prepared with agar (1%) equilibrated at 42°C. On top of the agar layers conditioned media from COS-7 cells stably transfected with a SLIT2 expression vector, or mock transfected control, was added. After 14 days of incubation, the colonies (>50 cells) were counted using an inverted microscope. All experiments were done at least three times in triplicate per experimental point and all statistical analyses were performed using the Student's *t*-test.

### Expression profiling analysis of the normal-tumour cell co-culture experiment

Genes differentially expressed were first identified using SAM multi-class test [22], with a threshold of FDR<1%. Differentially expressed genes were then used as input for principal component analysis (PCA) and the first two components representing 68% of variability were plotted to visualize the relationships between the different samples (**Fig 5A**). Genes differentially expressed in a given cell type as a result of co-culture were identified by a 2-class SAM procedure (FDR<1%) by directly comparing RWPE1 co-cultured with DU145 (RWPE1_DU145_) and RWPE1 cultured in isolation (RWPE1) or by comparing DU145 co-cultured with RWPE1 (DU145_RWPE1_) and DU145 cultured in isolation (DU145).

Predicted targets of polarized genes and the differentially expressed genes were then compared using a Fisher exact test. The comparison of these gene lists at the functional level was performed by plotting the frequency of genes in each functional term for predicted targets (x axis in **Fig 5C and 5D**) against differentially expressed genes (y axis in **Fig 5C and 5D**).

### Statistical modelling linking genetic mutations, transcription of polarised genes, tumour features and clinical outcome

In order to address the hypothesis that disease linked genetic mutations such as copy number variation (CNV) may influence the expression of polarised genes in tumour cells and that this, in turn, may be predictive of tumour features and clinical outcome we implemented a data analysis pipeline based on a number of advanced statistical procedures.

We used an independent dataset [17], which had comparative genomic hybridisation (CGH), gene expression and comprehensive information on tumour features and clinical outcome for a total of 231 tumour samples.

Firstly, in order to prioritise relevant genetic abnormalities we used ANOVA to rank CGH signals linked to tumour features and/or one of the clinical outcome variables (see **S1 File** for further details). The top 2017 probes in the ranked list were selected as an input of the modelling procedure.

We then mapped the 391 polarised genes we originally identified on the independent dataset. Next, we used the selected CGH data and the polarised gene expression profiling dataset as an input of a hierarchical Bayesian model [55] to find association between polarized gene expressions and CNV (see **S2 File** for details of the modeling procedure as applied here). Next, we fit an ANCOVA model for each gene expression on the tumour features. We then computed correlations for the significant associations (p<0.05) and integrated all information in a network format using the Cytoscape [43] software (**Fig 6**). Finally, we selected all polarized genes represented in the network and performed a survival analysis testing the hypothesis that their expression in tumour cells could be linked to clinical outcome (survival and time free of recurrence).

Survival analysis was performed as below. Briefly, for each gene we defined an optimal cutoff to separate patients in two groups of low and high-expressing tumours, using procedure described in Budczies *et al* [56]. Using this cut off, we dichotomized each gene expression level that was then used to fit a cox regression model.

## Supporting Information

S1 Fig Distribution of Spearman correlation coefficient in the Singh dataset. The distribution of the gene-to-gene Spearman correlation coefficient between genes expressed in the normal, tumour and between genes expressed in normal and tumour tissues.
(TIF)

S2 Fig Statistical validation of the polarization index. (A) Flowchart of the procedure used to estimate the probability of observing polarized genes by random chance. Prostate normal and tumour gene expression data are independently used to derive two correlation matrixes representing the correlation structure within each of the tissues. (B) Using these correlation matrixes as an input of a Multivariate Gaussian model we simulated synthetic normal and tumour datasets and finally we compute the polarization coefficient using these data.
(TIF)

S3 Fig Functional analyses of the targets of polarized genes. The Venn diagram lists functional terms significantly enriched in the targets of polarized genes. The diagram shows terms in common (red) as well as specifically enriched in positively and negatively polarized genes.
(TIF)

S4 Fig Comparison between targets of polarized genes and experimental cell communication transcriptional signatures. The gene level overlap between predicted and experimental cell-to-cell communication signatures. The two Venn diagrams show the comparison between the targets of (A) positively or (B) negatively polarized genes and the list of up and down-regulated differentially expressed genes in the *in vitro* cell communication model. Normal_T_ are genes differentially expressed in normal cells as a result of co-culture with tumour cells; Tumour_N_ are genes differentially expressed in tumour cells as a result of co-culture with normal cells.
(TIF)

S5 Fig Slit 2 expression in normal and tumour cells. (TIF)

S6 Fig Gene re-expression following treatment with hypomethylating agents and polarization. The frequency plot shows the distribution of the polarization index. The plot below shows the genes that are re-expressed as a result of exposure to hypomethylating agents. Note that genes are enriched in the positive end of the distribution (pol>0.75, p<0.03).
(TIF)

S7 Fig Survival analyses. (A) The distribution of *p values* from a cox model linking expression of polarized genes and survival free of recurrence. It compares genes that are linked to both CNV and Gleason score (CNV^+^_AND_ Gle^+^) with genes that are linked to Gleason score but not to CNV (CNV^-^_AND_ Gle^+^) and finally to all genes linked to CNV (CNV^+^). Note that genes linked to CNV and Gleason score have a higher association with survival respect to genes that are linked to Gleason score and not to CNV. (B) shows the–log10 of the p value (bar plot on the right side) and the value of beta parameter (bar plot on the left side) for the of the Cox survival model. The red dotted line shows the p<0.01 threshold of significance. Note that a negative beta means that higher expression of the gene in question has a lower hazard risk (higher chance of survival).
(TIF)

S8 Fig A high frequency of polarized genes does not occur as a result of experimental noise. (A-B) The original dataset from normal cells is used to add noise depending on signal levels (shown in panel B) multiplied by a scaling factor γ. The observed levels of polarization index computed from these synthetic datasets is therefore due to random experimental noise. (C) The number of highly polarized genes in the Singh *et al* dataset and synthetic dataset across the distribution of correlations. Note that the shape of the distribution of correlations between the real and synthetic dataset. (D) The distribution of polarization index between the Singh *et al* and synthetic datasets.
(TIF)

S9 Fig Distribution of the polarization coefficient in the kidney and liver datasets. The figure shows the distribution of polarization coefficient for the (A) kidney and the (B) liver gene expression profiling datasets. (C) For reference purposes the distribution of the polarization coefficient for the prostate cancer dataset is shown.
(TIF)

S1 Table Functional analysis of genes in the NT network modules. The excel file is the output of the web based tool g-profiler for genes in modules 1, 2 and 3 (see **Fig 1**).
(XLSX)

S2 Table Polarized genes. The table represents the full list of polarized genes with their polarization index (pol), the number of genes connected to each polarized gene in tumour (f) and in normal (b) cells.
(XLSX)

S3 Table Functional analysis of polarized genes. The excel file is the output of the web based tool DAVID for positively and negatively polarized genes.
(XLSX)

S4 Table Targets of polarized genes. The excel spreadsheet shows the list of genes connected to positively or negatively polarized genes.
(XLSX)

S5 Table Functional analysis of target genes. The excel file is the output of the web based tool DAVID for the targets of positively and negatively polarized genes.
(XLSX)

S6 Table Genes known to be re-expressed in prostate cancer cells, following exposure to hypomethylating agents. (XLSX)

S7 Table Clinical variables available in the Taylor *et al* dataset. (XLSX)

S8 Table Correlation between CGH and expression in polarized genes. The table shows the correlation coefficients between CGH signal and gene expression for all polarized genes. Note that only nine genes show a statistically significant correlation at a p<0.01.
(XLSX)

S9 Table Results of the Bayesian hierarchical model. The table shows posterior probabilities of inclusion for the significant associations between polarized genes and CGH probes. The rightmost columns show the chromosomal location of the CGH probes.
(XLSX)

S10 Table Functionally annotated CNV genes affecting the expression of polarized genes. (XLSX)

S11 Table Results of ANCOVA linking polarized genes to tumour features. The table shows correlations between polarized genes and tumour features. We fit an ANCOVA model for each gene expression on the tumour features. Correlations are computed only if the corresponding association is significant (p<0.05), otherwise they are set to zero.
(XLSX)

S12 Table Survival analysis. The table shows the results of the survival analysis on the polarized genes significantly associated with either the Gleason score or CNV. More specifically, for each gene it shows the polarization direction, the estimate of the coefficient of a cox regression model, the corresponding p-value and the corrected p-value after the Benjamini and Hochberg correction. For each gene an optimal cutoff was defined to separate patients in two groups of low and high-expressing tumours. The resulting dichotomized gene expression levels were then used to fit the cox regression model. The rightmost columns show whether the gene is associated with Gleason score, CNV or both. The analysis is performed twice on survival and time of recurrence.
(XLSX)

S1 File Details on data processing for the public domain microarray datasets. (DOCX)

S2 File Details of the hierarchical Bayesian model used to find associations between polarized gene expressions and Copy Number Variations (CNV). (DOCX)
