# Correction: A Network Biology Approach Identifies Molecular Cross-Talk between Normal Prostate Epithelial and Prostate Carcinoma Cells

**DOI:** 10.1371/journal.pcbi.1005172

**Published:** 2016-10-17

**Authors:** The PLOS Computational Biology Staff

There is an error in the author list. The author name ‘Philipp Antzack’ should read ‘Philipp Antczak’.

The article citation should read as follows:

Trevino V, Cassese A, Nagy Z, Zhuang X, Herbert J, Antczak P, et al. (2016) A Network Biology Approach Identifies Molecular Cross-Talk between Normal Prostate Epithelial and Prostate Carcinoma Cells. PLoS Comput Biol 12(4): e1004884. doi:10.1371/journal.pcbi.1004884
