# Supplementary figures and images for: A Network Biology Approach Identifies Molecular Cross-Talk between Normal Prostate Epithelial and Prostate Carcinoma Cells
Source: PLoS Comput Biol. 2016 Apr 28;12(4):e1004884. doi: 10.1371/journal.pcbi.1004884 (PMC4849722; doi:10.1371/journal.pcbi.1004884)

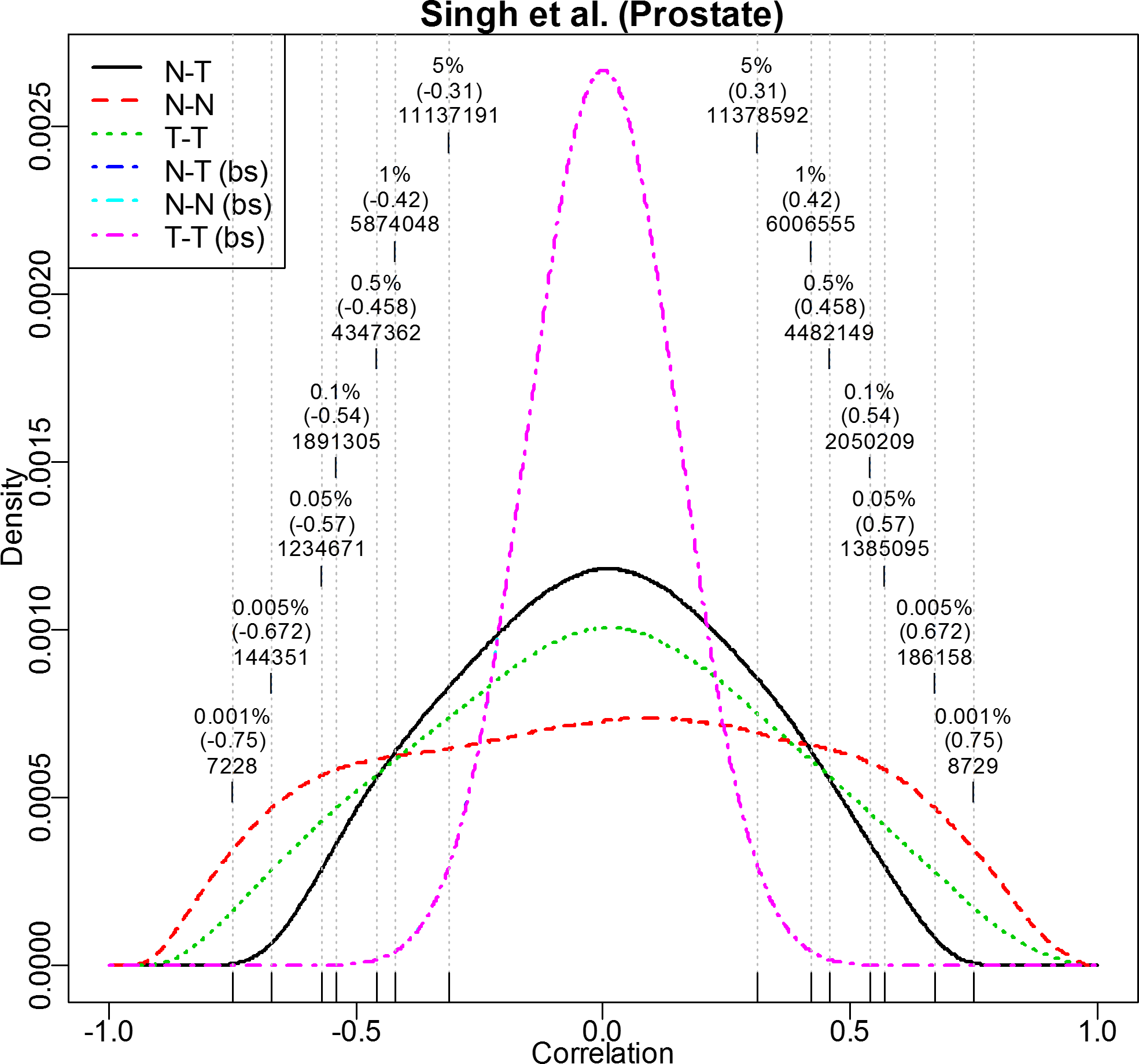

Supplement: S1 Fig — The distribution of the gene-to-gene Spearman correlation coefficient between genes expressed in the normal, tumour and between genes expressed in normal and tumour tissues. (TIF) [file pcbi.1004884.s001.tif]

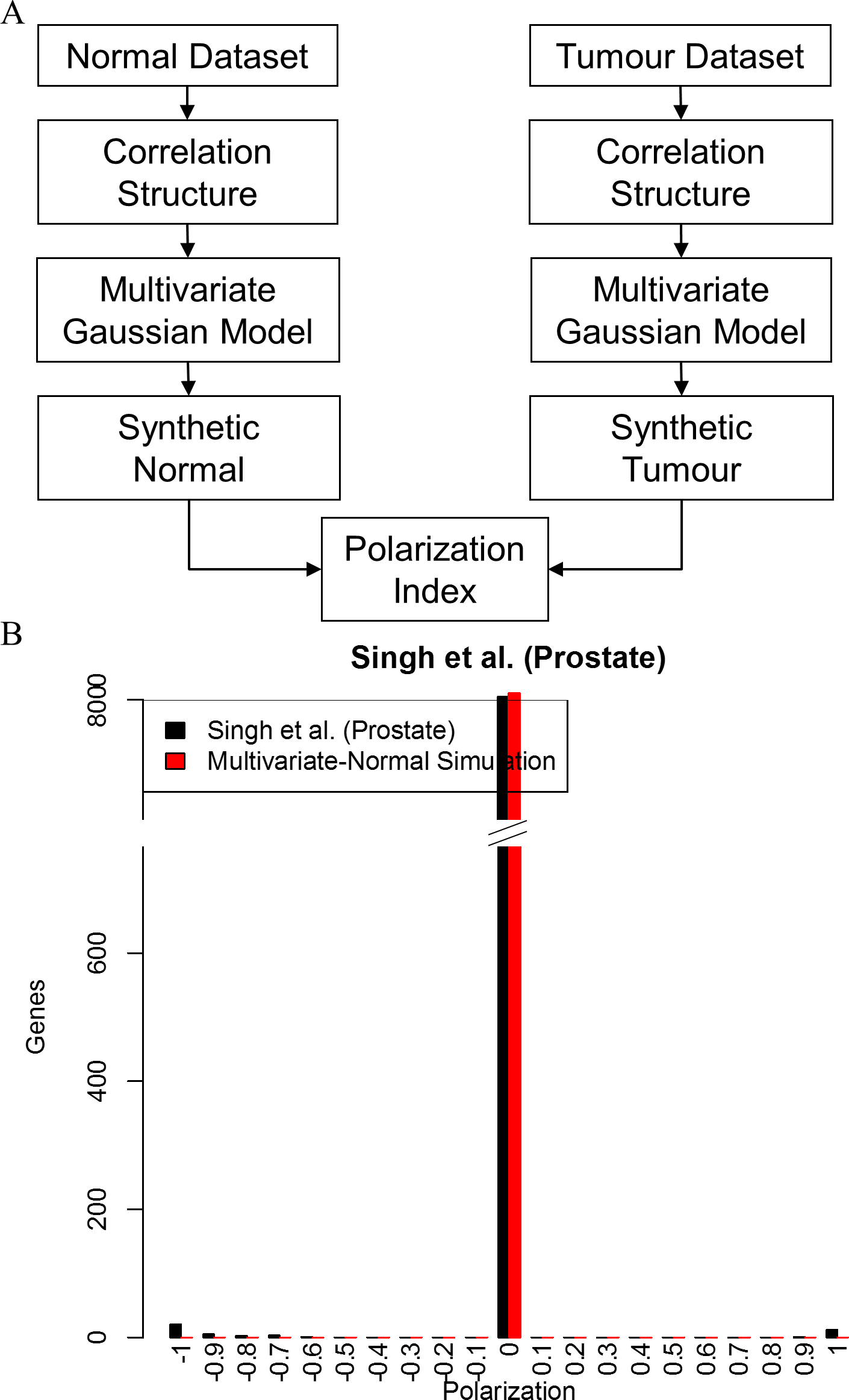

Supplement: S2 Fig — (A) Flowchart of the procedure used to estimate the probability of observing polarized genes by random chance. Prostate normal and tumour gene expression data are independently used to derive two correlation matrixes representing the correlation structure within each of the tissues. (B) Using these correlation matrixes as an input of a Multivariate Gaussian model we simulated synthetic normal and tumour datasets and finally we compute the polarization coefficient using these data. (TIF) [file pcbi.1004884.s002.tif]

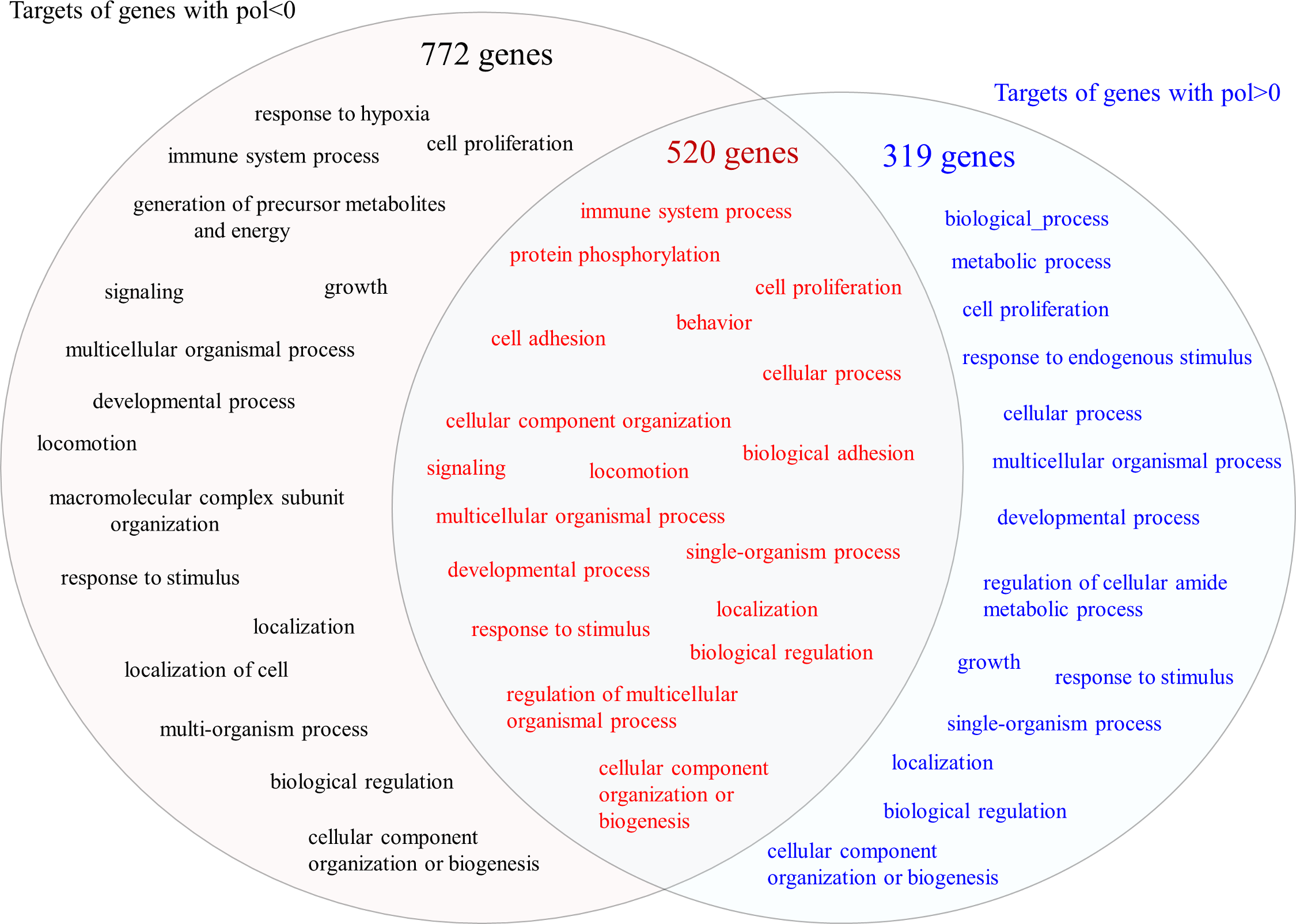

Supplement: S3 Fig — The Venn diagram lists functional terms significantly enriched in the targets of polarized genes. The diagram shows terms in common (red) as well as specifically enriched in positively and negatively polarized genes. (TIF) [file pcbi.1004884.s003.tif]

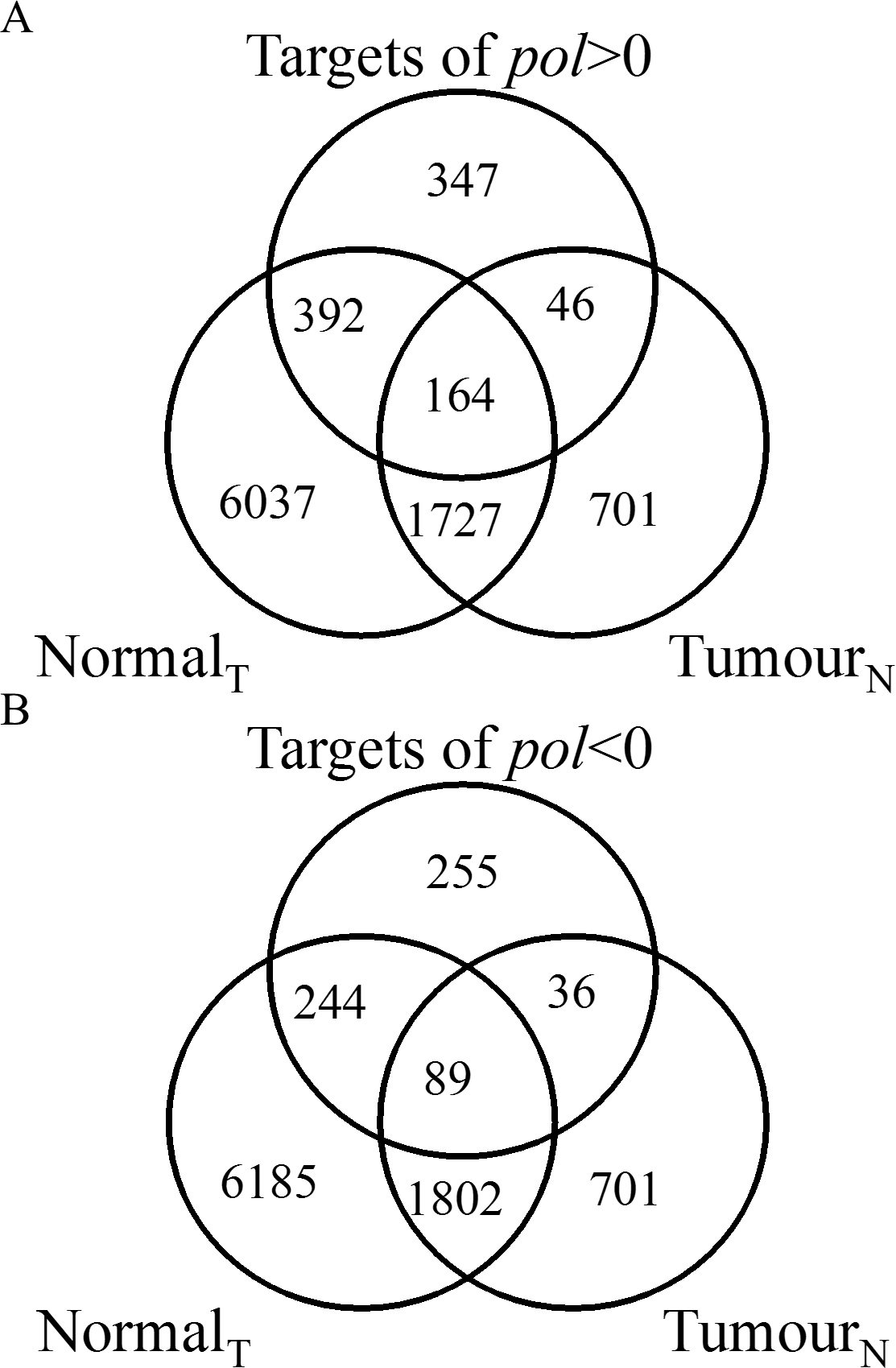

Supplement: S4 Fig — The gene level overlap between predicted and experimental cell-to-cell communication signatures. The two Venn diagrams show the comparison between the targets of (A) positively or (B) negatively polarized genes and the list of up and down-regulated differentially expressed genes in the in vitro cell communication model. NormalT are genes differentially expressed in normal cells as a result of co-culture with tumour cells; TumourN are genes differentially expressed in tumour cells as a result of co-culture with normal cells. (TIF) [file pcbi.1004884.s004.tif]

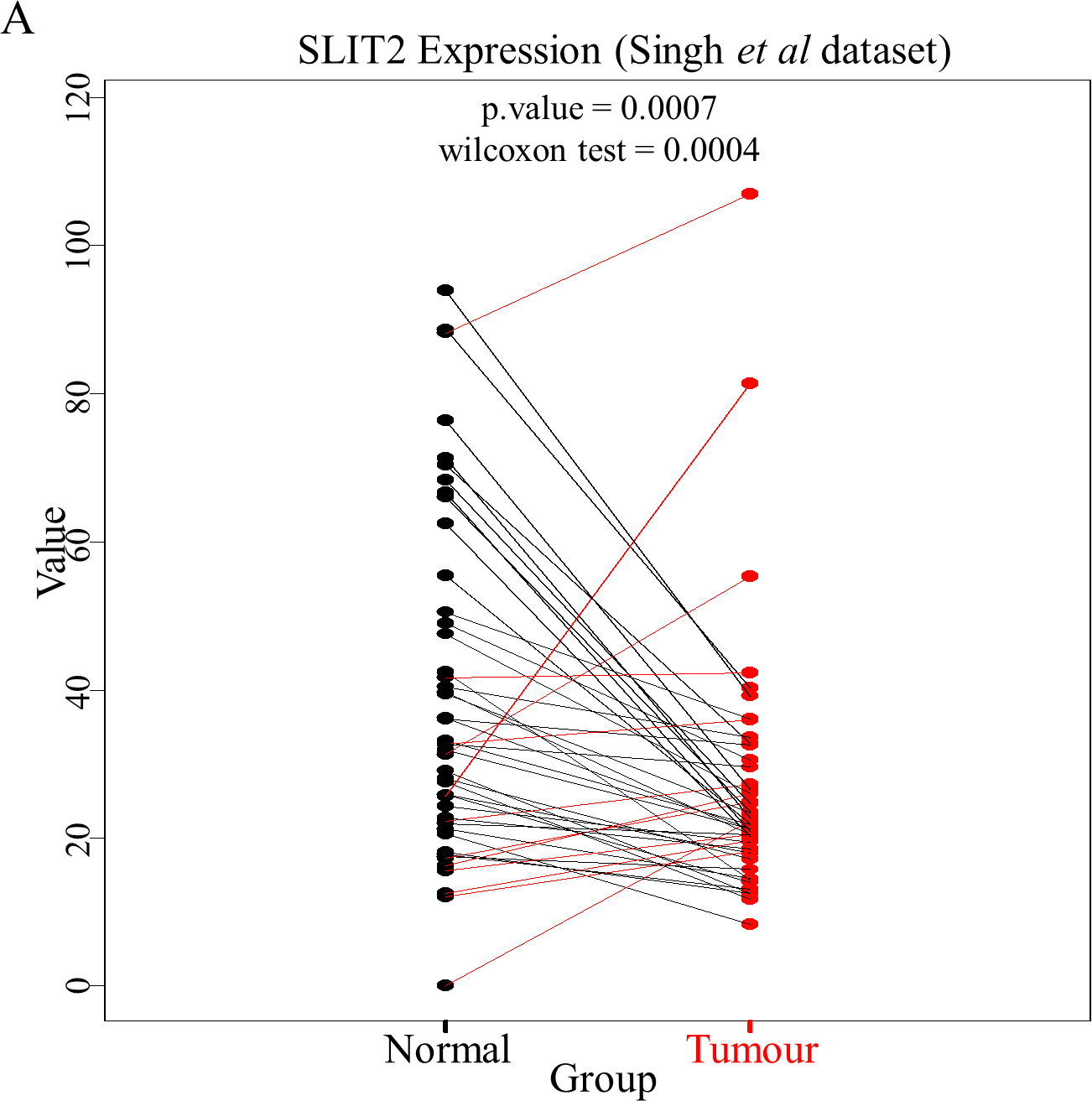

Supplement: S5 Fig — (TIF) [file pcbi.1004884.s005.tif]

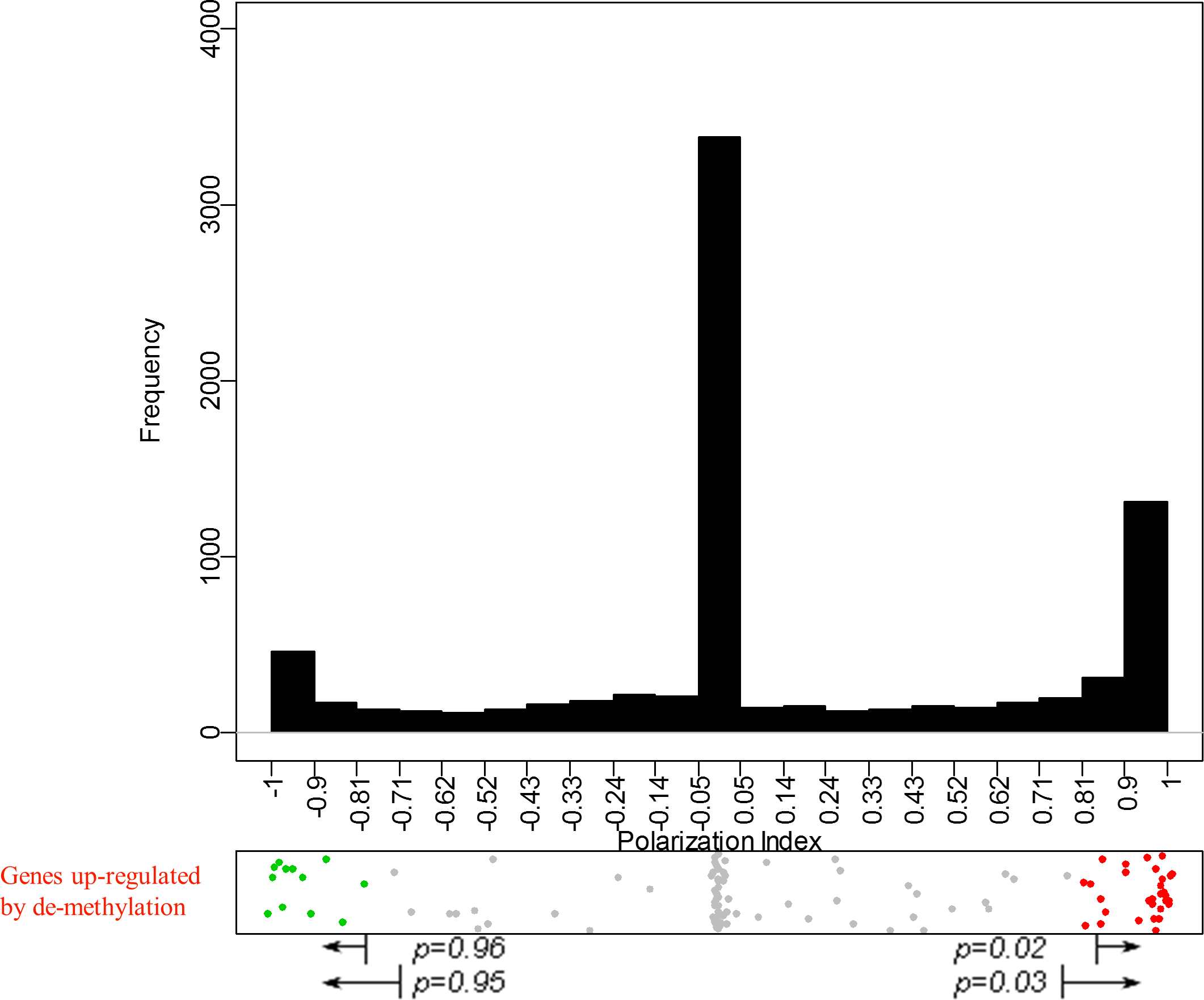

Supplement: S6 Fig — The frequency plot shows the distribution of the polarization index. The plot below shows the genes that are re-expressed as a result of exposure to hypomethylating agents. Note that genes are enriched in the positive end of the distribution (pol>0.75, p<0.03). (TIF) [file pcbi.1004884.s006.tif]

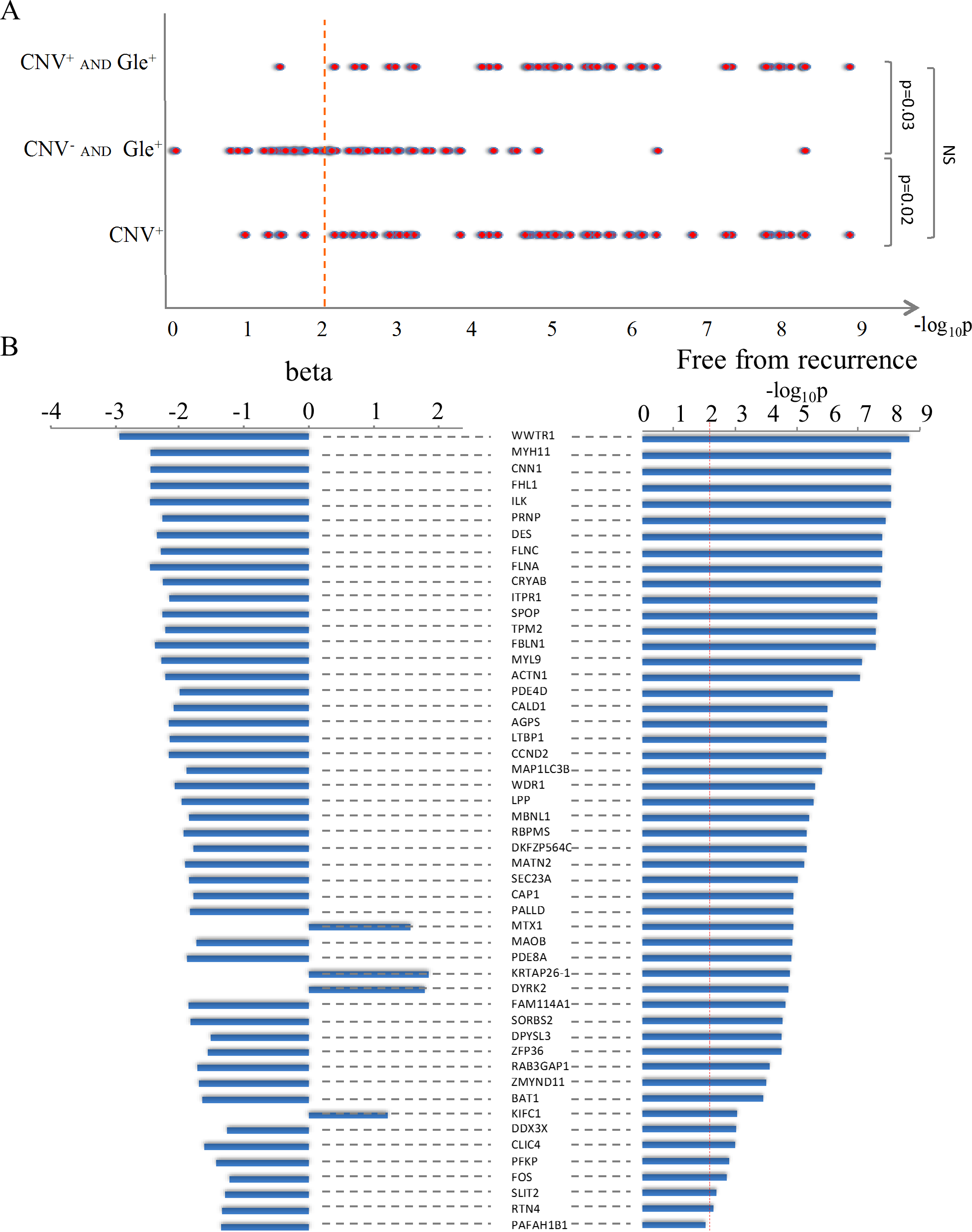

Supplement: S7 Fig — (A) The distribution of p values from a cox model linking expression of polarized genes and survival free of recurrence. It compares genes that are linked to both CNV and Gleason score (CNV+AND Gle+) with genes that are linked to Gleason score but not to CNV (CNV- AND Gle+) and finally to all genes linked to CNV (CNV+). Note that genes linked to CNV and Gleason score have a higher association with survival respect to genes that are linked to Gleason score and not to CNV. (B) shows the–log10 of the p value (bar plot on the right side) and the value of beta parameter (bar plot on the left side) for the of the Cox survival model. The red dotted line shows the p<0.01 threshold of significance. Note that a negative beta means that higher expression of the gene in question has a lower hazard risk (higher chance of survival). (TIF) [file pcbi.1004884.s007.tif]

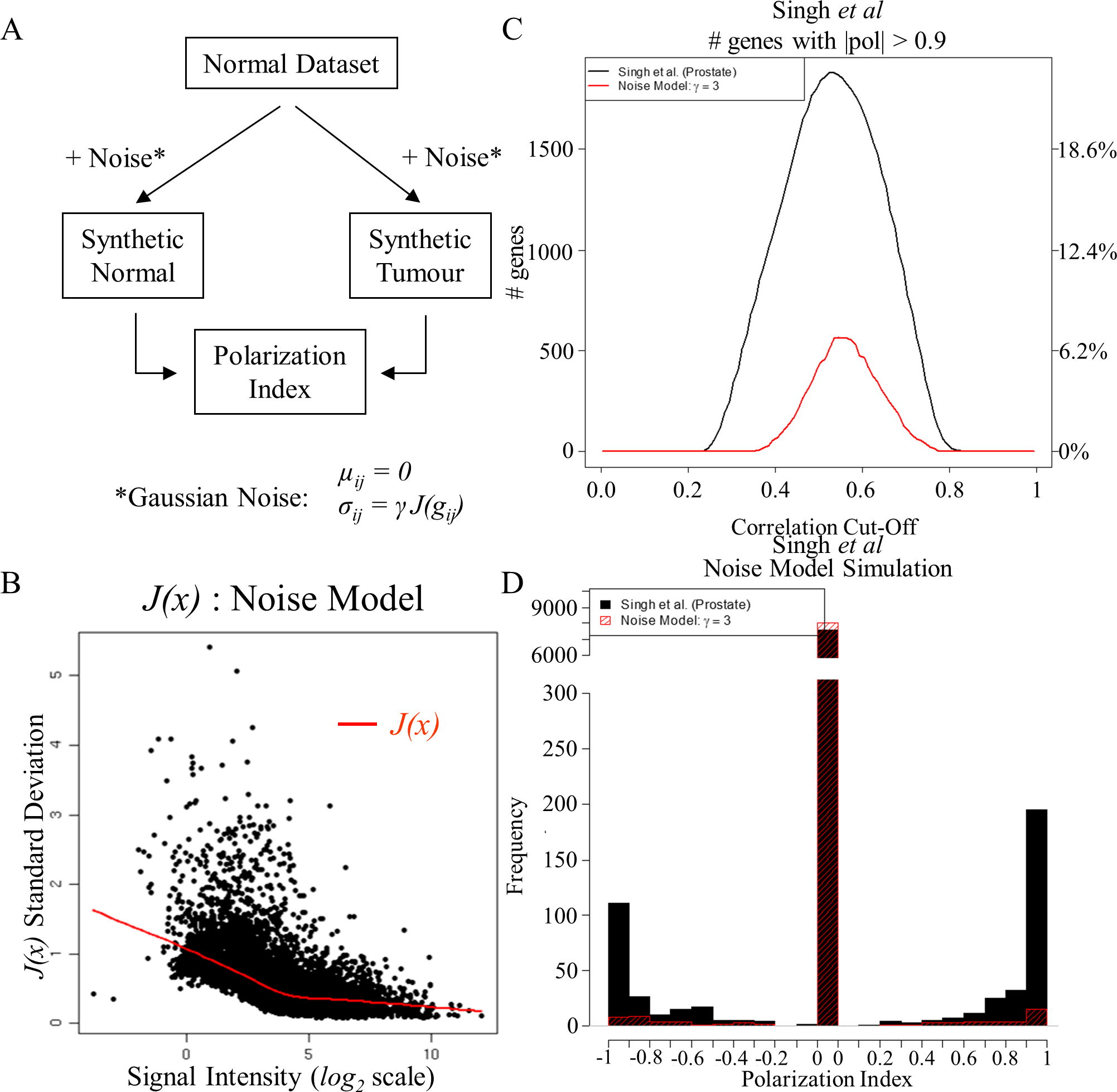

Supplement: S8 Fig — (A-B) The original dataset from normal cells is used to add noise depending on signal levels (shown in panel B) multiplied by a scaling factor γ. The observed levels of polarization index computed from these synthetic datasets is therefore due to random experimental noise. (C) The number of highly polarized genes in the Singh et al dataset and synthetic dataset across the distribution of correlations. Note that the shape of the distribution of correlations between the real and synthetic dataset. (D) The distribution of polarization index between the Singh et al and synthetic datasets. (TIF) [file pcbi.1004884.s008.tif]

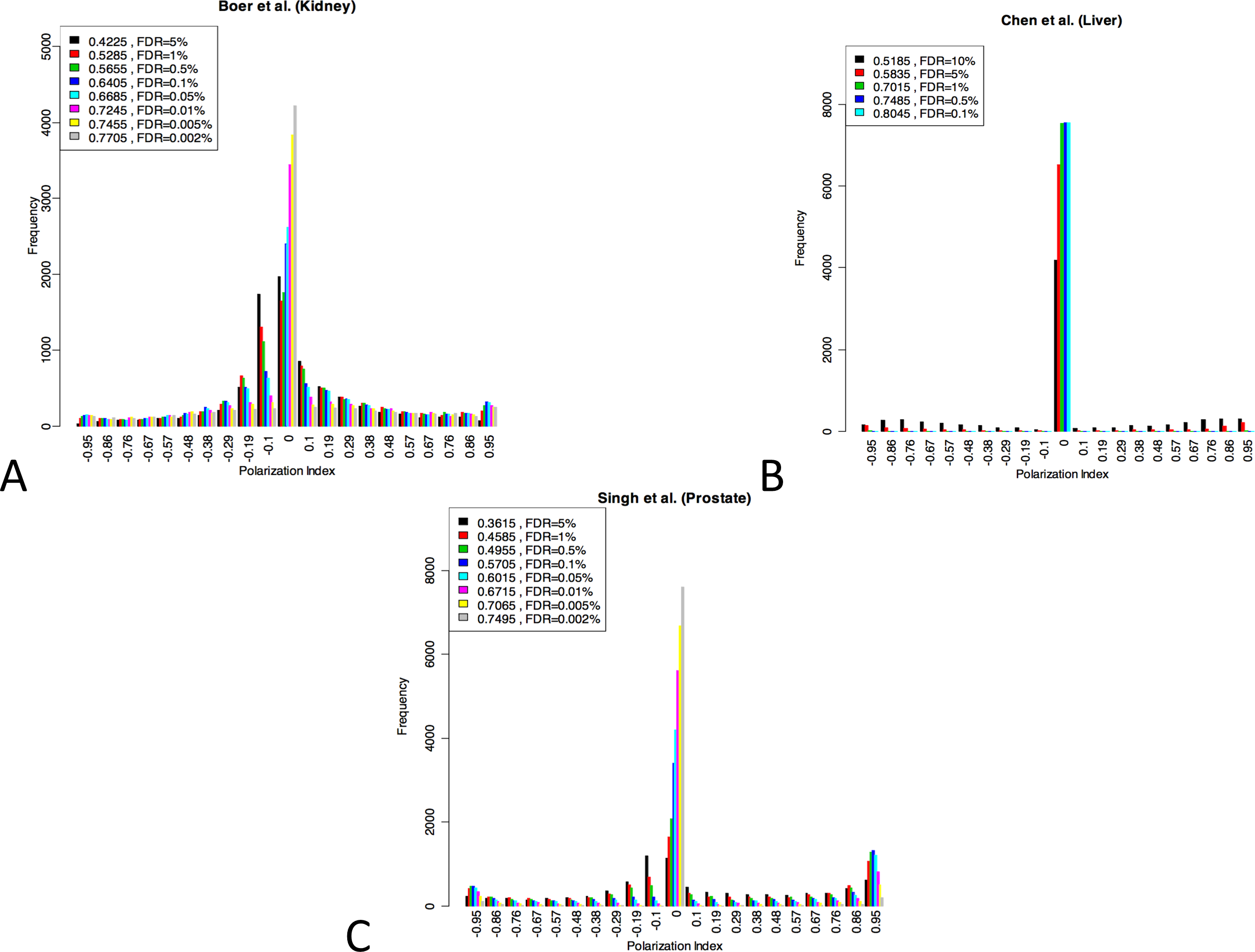

Supplement: S9 Fig — The figure shows the distribution of polarization coefficient for the (A) kidney and the (B) liver gene expression profiling datasets. (C) For reference purposes the distribution of the polarization coefficient for the prostate cancer dataset is shown. (TIF) [file pcbi.1004884.s009.tif]
