# Supplementary material for: A Network Biology Approach Identifies Molecular Cross-Talk between Normal Prostate Epithelial and Prostate Carcinoma Cells
Source: PLoS Comput Biol. 2016 Apr 28;12(4):e1004884. doi: 10.1371/journal.pcbi.1004884 (PMC4849722; doi:10.1371/journal.pcbi.1004884)
Supplement: S1 File — (DOCX) [file pcbi.1004884.s022.docx]

**Extended View File 1**

**Singh *et al.* data pre-processing.** The gene expression dataset was downloaded from original location (<http://www-genome.wi.mit.edu/MPR/prostate>). Unpaired samples were removed. The original data was transformed by a generalized logarithm function then quantile normalized. Low expressed genes were removed if the mean was less than 1. The used dataset contained 47-paired tumor-normal samples and 8,079 genes (67%).

**Taylor *et al.* data pre-processing.** The copy number variation dataset were downloaded from <http://cbio.mskcc.org/cancergenomics/prostate/data/> and GSE21035 from GEO at NCBI. The data was reduced to 1/10^th^ of the original size by smoothing the original signal ordered by chromosomal position using a running median of 3 consecutive probes then taking 1 smoothed probe every 10 probes. The used dataset contained 231 tumor samples by 24,355 probes.
