# Supplementary material for: A Network Biology Approach Identifies Molecular Cross-Talk between Normal Prostate Epithelial and Prostate Carcinoma Cells
Source: PLoS Comput Biol. 2016 Apr 28;12(4):e1004884. doi: 10.1371/journal.pcbi.1004884 (PMC4849722; doi:10.1371/journal.pcbi.1004884)
Supplement: S2 File — (DOCX) [file pcbi.1004884.s023.docx]

**Association between Gene Expression and Copy Number Aberration**

We employ a hierarchical Bayesian model to find associations between polarized gene expressions and Copy Number Variations (CNV). The model is fully described in Cassese et al. (2013). The method relates gene expression levels with CNV data, accounting for measurement error in the observed CGH intensities, via a Hidden Markov Model (HMM).

Denote with $Z=\left[Y,X\right]$the matrix of observed measurements and let$\xi=\left[\xi_{1},\ldots,\xi_{M}\right]$be the matrix of latent copy number states. We consider four ordered possible levels

$\xi_{im}=1$for copy number loss;

$\xi_{im}=2$for copy-neutral state;

$\xi_{im}=3$for a single copy gain;

$\xi_{im}=4$for multiple copy gains,

and assume the CGH probes ordered according to their chromosomal location. Our hierarchical model formulation treats CGH intensities as surrogates for the unobserved copy number states and can be expressed as

$$f\left(Z|\xi\right)=\prod_{i=1}^{n} \left\{\prod_{g=1}^{G} f(Y_{ig}|\xi_{i})\prod_{m=1}^{M} f(X_{im}|\xi_{im})\right\},$$

where the gene expression levels are regressed on hidden states,

$$Y_{ig}=\mu_{g}+\xi_{i}\beta_{g}+\varepsilon_{ig},$$

and where, conditional on the latent copy number states, the observed CGH measurements are assumed independent and normally distributed as

$$X_{im}|\left(\xi_{im}=j\right)\sim^{iid} N\left(\eta_{j},\sigma_{j}^{2}\right),$$

with$\eta_{j}$ and $\sigma_{j}^{2}$ representing the expected $\log_{2}$ ratio and the variance of all CGH probes in state *j* (*j*=1,…,4). Finally, the state persistence feature of CNV data is captured by a first order Markov model, which assumes that the probability of being in a particular copy number state, for a given probe, depends only on the state assigned to the previous one,

$$P\left(\xi_{i(m+1)}|\xi_{i1},\ldots,\xi_{im}\right)=P\left(\xi_{i(m+1)}|\xi_{im}\right)=a_{\xi_{im}\xi_{i(m+1)}},$$

with$A=\left(a_{hj}\right)$ the matrix of transition probabilities shared across chromosomes.

We employ a variable selection approach, introducing a binary matrix $R$ which encodes the network of gene-CGH association, and imposing spike and slab priors on the regression coefficients $\beta$, see George and McCulloch (1997)

$$\pi\left(\beta_{gm}|r_{gm},\sigma_{g}^{2}\right)=r_{gm}N\left(c^{-1}\sigma_{g}^{2}\right)+\left(1-r_{gm}\right)\delta_{0}\left(\beta_{gm}\right),$$

with$\delta_{0}$ a point mass at zero and $c>0$a hyperparameter to be chosen. We impose a Gamma prior on$\sigma_{g}^{-2}$ and a Normal distribution on $\mu_{g}|\sigma_{g}^{2}$. On the elements of the $R$ matrix we develop a prior that explicitly encourages close probes with common CNV structure to assume the same value

$$\pi\left(r_{gm}|rest\right)=\gamma_{m}\left[\pi_{1}^{r_{gm}}\left(1-\pi_{1}\right)^{1-r_{gm}}\right]+\sum_{j=1}^{2} \omega_{m}^{(\mathrm{j})}I_{\left\{r_{gm}=r_{g(m+(-1)^{\mathrm{j}})}\right\}},$$

with the constrains$\gamma_{m}\in\left[0,1\right]$and $\sum_{j=1}^{2} \omega_{m}^{(\mathrm{j})}=\left(1-\gamma_{m}\right)$. The probe-specific parameters are defined as

$$\gamma_{m}=\frac{\alpha}{\alpha+s_{(m-1)m}+s_{m(m+1)}},$$

$$\omega_{m}^{(1)}=\frac{s_{(m-1)m}}{\alpha+s_{(m-1)m}+s_{m(m+1)}},$$

$$\omega_{m}^{(2)}=\frac{s_{m(m+1)}}{\alpha+s_{(m-1)m}+s_{m(m+1)}},$$

with$\alpha$ set to a positive real value. The role of these parameters is to capture information on the physical distance between CGH probes and their unobserved copy number states, and this is done defining

$$s_{(m-1)m}=\frac{1}{n}\sum_{i=1}^{n} \frac{e^{\{1-\frac{d_{m}}{D}\}}-1}{e-1} I_{\{\xi_{im}=\xi_{i(m-1)}\}},$$

where$d_{m}$ is the distance between adjacent probes and $D$ a fixed quantity.

For posterior inference, we design a Markov Chain Monte Carlo (MCMC) stochastic search variable selection algorithm, composed by the following steps:

• Update $\mathbf{R}$ with Add/Delete or Swap moves; for Add/Delete select at random one of the elements in a row of $\mathbf{R}$ and change its value; for Swap select two elements with opposite status and swap their values. CGH probes with more than $n\times p_{MC}$ samples called in copy neutral state are not considered as potential association.

• Update $\mathbf{\xi}$ by choosing one column at random, and use it for all selected rows. Propose a new value by sampling from the transition matrix $\mathbf{A}$.

• Update the state specific means and variances via Gibbs steps.

• Update the transition matrix $\mathbf{A}$ via a Metropolis step, where each row is proposed by sampling from a Dirichlet distribution.

Final inference is done based on the output of the MCMC algorithm described above. For each element of the association matrix $\mathbf{R}$, its marginal posterior probability of inclusion (PPI) is estimated by counting the number of iterations where that element was set to 1. One can then select the most relevant associations by choosing a threshold and selecting those that have a PPI greater than the threshold. Finally, each element of $\mathbf{\xi}$ is estimated by calculating the most frequent state value.

Results we report in the paper were obtained with the following hyperparameter settings. As for the parameters that controls shrinkage of the model, we set $c=10$ assuming $\mu_{g}|\sigma_{g}^{2}\sim N(0,c_{\mu}^{-1}\sigma_{g}^{2})$ with $c_{\mu}=10^{-6}$. Moreover, we imposed a Gamma prior on the error precision, $\sigma_{g}^{-2}\sim G(\frac{\delta}{2},\frac{d}{2})$, set $\delta=3$ and chose $d$ such that the expected value of the gene specific variance represents 5% of the observed variance of the standardized responses. We set the expected a priori probability of a link to be included in the model to 1%, by imposing a Beta(.1,.99) prior on $\pi_{1}$ (in the posterior derivations, this parameter was integrated out). Moreover, we set $\alpha=20$. For the HMM model, we set $\eta_{j}\sim N(\delta_{j},\tau_{j}^{2})\cdot\mathbf{I}_{\{low_{\eta_{j}}<\eta_{j}<upp_{\eta_{j}}\}}$,$\sigma_{j}^{-2}\sim Ga(b_{j},l_{j})\cdot\mathbf{I}_{\{\sigma<upp_{\sigma_{j}}\}}$, with $b_{j}=1$, $l_{j}=1$, $j=1,\ldots,4$, and the other hyperparameters specified as in the following Table:

| Param. | State 1 | State 2 | State 3 | State 4 |
| --- | --- | --- | --- | --- |
| $\delta_{j}$ | -1 | 0 | .58 | 1 |
| $\tau_{j}$ | 1 | 1 | 1 | 2 |
| $low_{\eta_{j}}$ | $-\infty$ | -.1 | .1 | $\eta_{3}+\sigma_{3}$ |
| $upp_{\eta_{j}}$ | -.1 | .1 | .73 | $\infty$ |
| $upp_{\sigma_{j}}$ | .41 | .41 | .41 | 1 |

We assumed independent Dirichlet priors across the rows of the transition matrix $\mathbf{A}$, and set all their hyperparameters to 1. We also set $D$ equal to the length of chromosome 8, and chose $p_{R}=.6$, $p_{\xi}=.3$, and $p_{MC}=.02$. Finally, when performing the final inference based on the output of the MCMC algorithm, we inspected the PPI plot and applied a posterior threshold equal to .5.

**References**

A. Cassese, M. Guindani, M.G.Tadesse, F. Falciani, and M. Vannucci. A hierarchical Bayesian model for inference of copy number variants and their association to gene expression. Annals of Applied Statistics, 8(1):148-175, 2014.

E. George and R.E. McCulloch. Approaches for Bayesian variable selection. Statistica Sinica, 7:339–373, 1997.
